# Enhancing crystal integrity and structural rigidity of CsPbBr_3_ nanoplatelets to achieve a narrow color-saturated blue emission

**DOI:** 10.1038/s41377-024-01441-1

**Published:** 2024-05-11

**Authors:** Qianqian Huang, Wenxu Yin, Bo Gao, Qingsen Zeng, Dong Yao, Hao Zhang, Yinghe Zhao, Weijia Zheng, Jiaqi Zhang, Xuyong Yang, Xiaoyu Zhang, Andrey L. Rogach

**Affiliations:** 1 Key Laboratory of Automobile Materials MOE, School of Materials Science & Engineering, and Jilin Provincial International Cooperation Key Laboratory of High-Efficiency Clean Energy Materials, Jilin University, Changchun, China; 2 State Key Laboratory of Supramolecular Structure and Materials, College of Chemistry, Jilin University, Changchun, China; 3 State Key Laboratory of Materials Processing and Die & Mould Technology, School of Materials Science and Engineering, Huazhong University of Science and Technology, Wuhan, Hubei China; 4 Department of Chemistry, University of Victoria, Victoria, BC Canada; 5 Key Laboratory of Advanced Display and System Applications of Ministry of Education, Shanghai University, Shanghai, China; 6 Department of Materials Science and Engineering, and Centre for Functional Photonics (CFP), City University of Hong Kong, Hong Kong S.A.R, China

**Keywords:** Lasers, LEDs and light sources, Photonic devices

## Abstract

Quantum-confined CsPbBr_3_ perovskites are promising blue emitters for ultra-high-definition displays, but their soft lattice caused by highly ionic nature has a limited stability. Here, we endow CsPbBr_3_ nanoplatelets (NPLs) with atomic crystal-like structural rigidity through proper surface engineering, by using strongly bound N-dodecylbenzene sulfonic acid (DBSA). A stable, rigid crystal structure, as well as uniform, orderly-arranged surface of these NPLs is achieved by optimizing intermediate reaction stage, by switching from molecular clusters to mono-octahedra, while interaction with DBSA resulted in formation of a Cs_x_O monolayer shell capping the NPL surface. As a result, both structural and optical stability of the CsPbBr_3_ NPLs is enhanced by strong covalent bonding of DBSA, which inhibits undesired phase transitions and decomposition of the perovskite phase potentially caused by ligand desorption. Moreover, rather small amount of DBSA ligands at the NPL surface results in a short inter-NPL spacing in their closely-packed films, which facilitates efficient charge injection and transport. Blue photoluminescence of the produced CsPbBr_3_ NPLs is bright (nearly unity emission quantum yield) and peaks at 457 nm with an extremely narrow bandwidth of 3.7 nm at 80 K, while the bandwidth of the electroluminescence (peaked at 460 nm) also reaches a record-narrow value of 15 nm at room temperature. This value corresponds to the CIE coordinates of (0.141, 0.062), which meets Rec. 2020 standards for ultra-high-definition displays.

## Abstract (web summary)

The chemical equilibrium is optimized using N-dodecylbenzene sulfonic acid for enhancing the crystal integrity and structural rigidity of CsPbBr_3_ perovskite nanoplatelets, resulting in their spectrally stable, bright and extremely narrow color-saturated blue emission.

## Graphical abstract

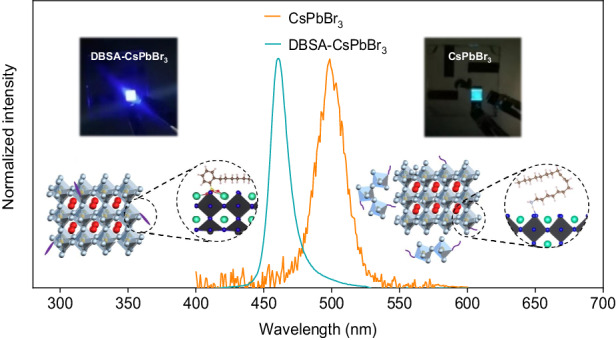

## Introduction

Display and energy-saving lighting applications require blue light-emitting diodes (LEDs) with the Commission Internationale de l’Eclairage (CIE) *y*-coordinate below 0.15, alongside with the (*x* + *y*)-value below 0.30^1^. Metal halide perovskites are potential candidates for meeting the requirements of ultra-high definition displays^2^ that possess color-saturated blue, green and red emission with CIE coordinates around (0.131, 0.046), (0.170, 0.797), and (0.708, 0.292), respectively, as they exhibit excellent optical properties, such as high photoluminescence quantum yield (PL QY), adjustable bandgap, high color purity, and wide color gamut, and can be produced by low-cost solution processing^3,4^. However, even though significant advances have been made in recent years towards red and green perovskite LEDs which are reaching close to 30% external quantum efficiencies (EQEs)^5–7^, it is still difficult to realize spectrally stable, color-saturated blue electroluminescence (EL) for perovskite LEDs^8^. Current efforts at achieving blue emission in perovskite LEDs largely rely on mixed-halide perovskite compositions suffering from ion migration-induced poor color stability^9,10^. Another way to tune the bandgap and the emission color of metal halide perovskites is by taking advantage of the quantum confinement effect while producing respective nanoparticles^11–14^, but this approach has its own disadvantages due to the reduced PL QYs caused by increased surface defects upon decreasing size, and poor stability of perovskites due to their low formation energy and ionic nature. Fortunately, surface engineering offers the possibility of passivating defects and obtaining more robust perovskite crystal structures. Surface engineering involves altering the chemical composition of the perovskite surface, which can be realized by introducing organic capping molecules or inorganic ligands^15–23^. On the one hand, this allows for the creation of highly sturdy surface bonding and an orderly arrangement of surface atoms, reducing defects and promoting radiative recombination. On the other hand, this can also enhance the overall structural rigidity of the perovskite crystals, which benefits from the surface strain generated by the ligands for nanostructures with large specific surface areas. There has been evidence that the phase transition point between different crystal phases varies greatly as a function of crystal size in lanthanide elements (e.g., NaYF_4_), oxides (e.g., TiO_2_), metals (e.g., Ag), and ferroelectrics (e.g., BaTiO_3_)^24–26^. Usually, the smaller the size, the higher the phase transition temperature. Similarly, taking bulk CsPbI_3_ as an example, the cubic *α*-phase with a direct bandgap is only stable at high temperatures (>633 K)^27^, and transforms into an orthorhombic phase with non-perovskite structure below 593 K^28^, whereas CsPbI_3_ nanoparticles can maintain their cubic phase below 273 K^28^. Thus, utilizing proper surface ligand engineering to optimize the surface structure of quantum-confined CsPbBr_3_ nanoparticles holds a great potential for achieving efficient and stable blue emitters^29^.

Here, we demonstrate how the crystal integrity and structural rigidity of quantum-confined color-saturated blue-emitting CsPbBr_3_ nanoplatelets (NPLs) can be significantly enhanced via realization of their ordered and stable surface structure using covalently bound molecule N-dodecylbenzene sulfonic acid (DBSA). These CsPbBr_3_ NPLs show near-unity PL QYs with strong color-saturated blue emission which is peaked at 457 nm PL with an extremely narrow bandwidth of 3.7 nm at 80 K. Blue-emitting LEDs based on these NPLs show stable EL peaking at 460 nm with a record narrow bandwidth of 15 nm at room temperature. This value corresponds to the CIE coordinates of (0.141, 0.062), which meets Rec. 2020 standards for ultra-high-definition displays.

## Results

### Synthesis of CsPbBr_3_ NPLs

According to the previous studies^30–32^, the surface of CsPbBr_3_ NPLs is terminated with bromide ions, and each oleylamine (OLA) ligand forms a hydrogen bond with Br^-^. We performed density functional theory (DFT) calculations which indicated that the bond energy between Pb^2+^ and Br^-^ significantly decreases from 2.08 to 1.21 eV after capping OLA-Br, leading to the formation of the vacancy defects and even structural collapse of the perovskite lattice when Br^-^ ions detach from the surface (Fig. S1). According to the DFT results, the adsorption energy between Pb^2+^ and deprotonated DBSA is 5.16 eV, which falls within the range of binding energies ranging from 3.1 to 8.2 eV for covalent bonds (Fig. S1), indicating that DBSA ligands are covalently bound due to the benzenesulfonic group’s high acidity and electron-withdrawing ability. Therefore, DBSA ligands can be used to achieve stable surface structures on CsPbBr_3_ NPLs, eventually able to prevent ligand desorption, inhibit ionic migration, and improve the crystal stability.

**Fig. 1:**
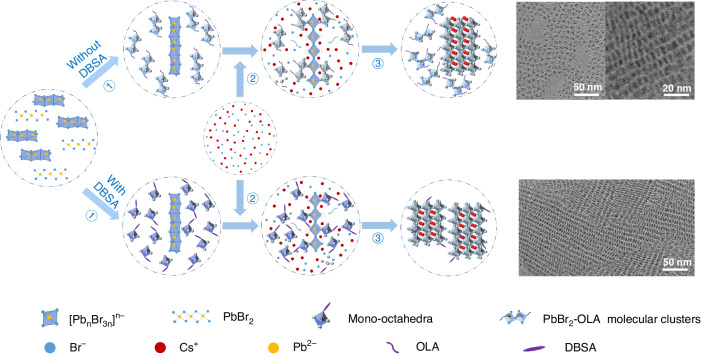
Growth mechanism analysis. Schematic illustration of the growth mechanism for CsPbBr_3_ NPLs synthesized without (upper part) and with the use of DBSA (bottom part), and their corresponding TEM images provided on the right

Perovskite nanocrystals can be synthesized using a variety of techniques, including ligand-assisted reprecipitation (LARP), hot injection, ultrasonication, microwaves, solvothermal, and saponification^33–35^. Among them, the LARP approach relies on changes in solubility of perovskite precursors^36,37^, while others require energy supplies, such as thermal or ultrasonic energy, to trigger the chemical reaction between the precursors. In this study, a modified LARP synthesis at room temperature has been employed to obtain CsPbBr_3_ NPLs capped with DBSA ligands (see Materials and methods for details). As a first step, PbBr_2_ dissolved in dimethyl sulfoxide (DMSO) is injected into a mixture solution containing oleic acid (OA), OLA, and toluene, with or without DBSA. When DBSA is absent in this mixture, PbBr_2_-OLA molecular clusters and [Pb_n_Br_3n_]^n-^ chains (n > 1) are formed, as illustrated in Fig. 1, upper part. This assumption follows from the pronounced absorption at 396 nm which belongs to molecular clusters^38^, while absorption ranging from 350 to 380 nm indicates the formation of [Pb_n_Br_3n_]^n-^ chains (Fig. S2), which is consistent with previous studies^39^. In contrast, when DBSA is present, there is no absorption peak at 396 nm (Fig. S2), suggesting that the formation of molecular clusters is inhibited due to the strong interaction between DBSA and Pb^2+^ (Fig. S1), resulting in the conversion of molecular clusters into smaller intermediate state structures, namely the mono-octahedra of PbBr_a_DBSA_b_^4-^ (a + b = 6), whose absorption extends into the ultraviolet region. At the same time, the absorption at 350–380 nm (Fig. S2) still indicates the presence of [Pb_n_Br_3n_]^n-^ chains, as illustrated in Fig. 1, bottom part. Also, the presence of DBSA does not cause any additional absorption peaks to appear (Fig. S2), suggesting that no new intermediates are generated in the reaction.

In the second step, the CsBr precursor is injected, which results in a Br-rich environment. In the more tightly arranged face-sharing octahedrons, Br^-^ ions have higher energies than in the corner-sharing octahedrons due to an enhanced repulsive force between the adjacent Pb^2+^. Pauling’s third rule states that octahedrons accommodating cations tend to share corners rather than faces to increase distances and reduce repulsive forces. Thus, this transforms the face-sharing [Pb_n_Br_3n_]^n-^ chains into corner-sharing [Pb_n_Br_5n_]^3n-^ octahedron chains^40^. In the presence of Cs^+^, these corner-sharing chains are able to adsorb surrounding molecular clusters or mono-octahedra on their sides, inducing continuous crystal growth to generate CsPbBr_3_ NPLs. Those mono-octahedra can serve as intermediate states more conveniently than molecular clusters, and they can be arranged more orderly on the NPL surface structure. In the absence of DBSA, as a result of the fast crystallization process, synthesized CsPbBr_3_ NPLs have rather poor crystallinity and low uniformity of size and shape, as shown by the transmission electron microscopy (TEM) images in Fig. 1 at the top right. In the presence of highly viscous DBSA, the diffusion of mono-octahedra is slow, causing slow crystallization which results in an improved crystallinity of the CsPbBr_3_ NPLs; they are now characterized by high uniformity of shape and thickness (bottom right of Fig. 1).

For the eventual large-scale synthesis of perovskites, a primary prerequisite is the preservation of emission efficiency, color purity, and crystal quality of the final materials. A demonstration of the scaled-up synthesis has been conducted in our laboratory using 10 times larger volume (50 mL) than in the standard lab-scale synthesis (5 mL). It is apparent from Fig. S3a and S3b that the emission efficiency of CsPbBr_3_ NPLs remains unchanged even when the reaction volume has been increased by 10 times. When compared to the lab-level synthesis, the almost identical absorption, PL spectra and crystal structure of the CsPbBr_3_ NPLs suggest that high color purity and crystallinity can indeed be maintained (Figs. S3c and 3d).

In addition, thermal stability and the detrimental influence of polar solvents are considered to be significant factors affecting the properties of perovskite nanocrystals^41^. To check on those factors, according to the previous research^42,43^, temperatures ranging from 288 to 328 K and 10 μL of water additives have been chosen, which can easily convert blue emission of perovskite nanocrystals into green emission. The PL spectra and XRD patterns of DBSA-CsPbBr_3_ NPLs synthesized under the mentioned conditions are not affected at all (Fig. S4), demonstrating the robustness of the DBSA engineering approach against the conditions tested. Besides of the robustness, large-scale synthesis of perovskites also requires consideration of the cost-effectiveness. The price of DBSA is ~29 $ kg^−1^, less than that of OA (87 $ kg^−1^) and OLA (50 $ kg^−1^). Therefore, replacing those two conventional ligands with DBSA to optimize the perovskite quality is in line with potential commercialization objectives. As a room-temperature synthesis method, another advantage of the DBSA engineering approach is that it does not require vacuum equipment, precise temperature control, or expensive inert gases to protect the reaction environment, making it suitable for producing high-quality perovskite nanocrystals on a large scale. Simple and flexible operation methods can significantly reduce instrument costs and production times, and thus this synthetic approach has significant commercialization potential.

### Surface chemical composition

**Fig. 2:**
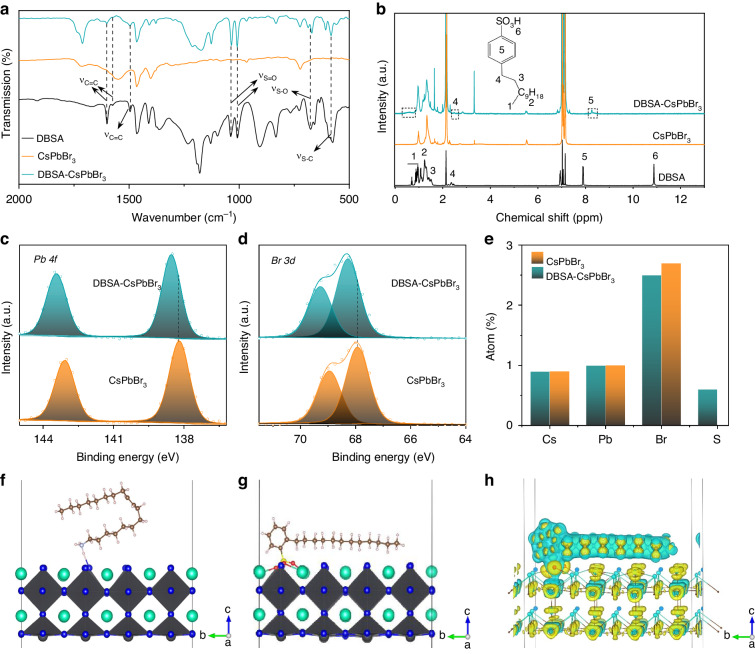
Surface chemical composition of CsPbBr_3_ and DBSA-CsPbBr_3_ NPLs. **a** FTIR and **b** 1H NMR spectra of DBSA, CsPbBr_3_ NPLs, and DBSA-CsPbBr_3_ NPLs. High-resolution XPS spectra of CsPbBr_3_ and DBSA-CsPbBr_3_ NPLs for **c** Pb *4f* and **d** Br *3d*. **e** Atomic composition (in terms of Cs, Pb, Br, and S elements) of CsPbBr_3_ and DBSA-CsPbBr_3_ NPLs determined from XPS data. DFT models of adsorption between **f** OLA-Br and **g** DBSA ligands and CsPbBr_3_ NPLs. **h** The charge density difference at the surface containing DBSA and Pb^2+^, with yellow color indicating an increase and blue color indicating a decrease in the electron density

To investigate their surface chemical composition, Fourier transform infrared spectroscopy (FTIR), 1H nuclear magnetic resonance (1H NMR), and X-ray photoelectron spectroscopy (XPS) measurements were conducted on purified CsPbBr_3_ NPLs synthesized with and without DBSA (CsPbBr_3_ NPLs produced with DBSA will be denoted as DBSA-CsPbBr_3_ NPLs in the forthcoming discussion, in order to distinguish them from the notation CsPbBr_3_ NPLs which will be used for the reference samples made without DBSA), which were obtained by centrifugation after adding methyl acetate. In the FTIR spectra provided in Fig. 2a, peaks of DBSA-CsPbBr_3_ NPLs at 1602, 1576, and 1495 cm^−1^ are associated with benzene stretching vibrations^44^; those peaks are absent in FTIR spectra of CsPbBr_3_ NPLs. Peaks at 1038, 1007, and 680 cm^−1^ seen in DBSA-CsPbBr_3_ NPLs belong to the S=O and S–O stretching vibrations of the sulfonate group (-SO_3_^-^)^45^, respectively, and the peak at 582 cm^−1^ is the tensile vibration of S–C^46^. The presence of all those characteristic vibration peaks indicates that the deprotonated DBSA is bound to the CsPbBr_3_ NPL surface. In the 1H NMR spectra shown in Fig. 2b, DBSA-CsPbBr_3_ NPLs possess five resonance peaks associated with DBSA containing six characteristic hydrogens, while the absence of resonance peak around 11 ppm indicates that the deprotonated sulfonic acid groups bind to Pb^2+^ at the DBSA-CsPbBr_3_ NPL surface. Furthermore, the prominent chemical shift towards the low-field, and a significant hydrogen peak broadening suggest a strong interaction between DBSA and CsPbBr_3_ NPLs^47^, which is also supported by the FTIR data (Fig. 2a). As judged from the FTIR and NMR spectra, the deprotonated sulfonic acid groups of DBSA are bound to the NPL surface, because DBSA has a greater bonding energy than OLA-Br. This is because DBSA is a soft X-type ligand, which has a strong affinity for binding to undercoordinated Pb^2+^ than hard X-type ligands such as OLA-Br, donating one electron to a halide anion.

XPS spectra further confirm the strong ligand-surface interaction in DBSA-CsPbBr_3_ NPLs, as opposed to the CsPbBr_3_ NPLs. Figure S5a shows the fitting for XPS peaks of S *2p* at 163.9 eV, 162.6 eV, and 161.3 eV, which correspond to S-C_6_H_4_-, S=O and S–O bonds, respectively. Figure S5b shows the fitting for XPS peaks of O *1s* at 531.7 eV and 529.4 eV corresponding to O–S and Pb–O, respectively; they match those in OA passivated perovskite nanocrystals^48^. Through the S–O–Pb bonds, DBSA ligands are tightly bound to the NPL surface. As compared to CsPbBr_3_ NPLs, the two distinctive peaks of Pb *4f* and Br *3d* are shifted to higher binding energy in DBSA-CsPbBr_3_ NPLs: from 143.1 and 138.2 eV to 143.4 and 138.6 eV for Pb *4f*, and from 68.9 and 67.9 eV to 69.3 eV and 68.3 eV for Br *3d*, respectively (Figs. 2c and 2d)^49^. This can be attributed to electron transfer from Pb^2+^ ions to ligands in DBSA-CsPbBr_3_ NPLs, due to the high adsorption energy between Pb^2+^ and deprotonated DBSA. As deducted from Fig. 2e, the atomic ratios of Pb-to-Br determined from XPS data is 1/2.7 in CsPbBr_3_ NPLs which is larger than the atomic ratio of Pb/(Br+S) equal to 1/3.3 in DBSA-CsPbBr_3_ NPLs (here, the S-content serves as an indicator of the amount of DBSA), suggesting that the uncoordinated Pb^2+^ have been fully passivated at the surface of the latter. All these data point out towards the formation of a stable and ordered surface structure in the case of in DBSA-CsPbBr_3_ NPLs, which enhances their structural rigidity.

A shift to higher binding energy in the XPS spectra of Cs *3d* in DBSA-CsPbBr_3_ NPLs is also detected (Fig. S5c), whereas conventional surface engineering for passivating surface Br-vacancy is not able to alter the bonding energy of Cs^+^ since these cations are located at the center of four [PbBr_6_]^4-^ octahedral, and thus do not participate in the orbital hybridization^50^. We employ DFT calculations (Fig. 2f, g) to identify the possible reason for the observed shift of the Cs *3d* XPS peaks. Unlike OLA ligands that only interact with one Pb^2+^ cation, DBSA can also form chemical bonds with three surrounding Cs^+^, with bond lengths of 2.91, 3.11, and 3.25 Å (Fig. S6), respectively, which is almost equivalent to the bonds formed in Cs_x_O, where the bond length is 2.86 Å^51^. Gain and lose of the electrons, and the electron density transfer between Cs and O atoms can be obtained from the Bader charge calculation (Table S1) and be seen from the charge density difference shown in Fig. 2h for DBSA-CsPbBr_3_ surface, which corresponds to the formation of chemical bonds. Thus, the surface of the DBSA-CsPbBr_3_ NPLs can be thought to be coated with a monolayer of Cs_x_O shell, which increases the overall Cs^+^ binding energy, stabilizes the surface and improves the structural rigidity of these NPLs.

Due to the multiple coordination between the DBSA ligands and the NPL surface (one Pb^2+^ and three Cs^+^) and the branched chains oriented parallel to the NPL surface, which is caused by ortho-substituted benzene of DBSA (Fig. 2g and Fig. S6b), DBSA-CsPbBr_3_ NPLs experience a lower ligand surface coverage as compared to CsPbBr_3_ NPLs, and thus show a shorter spacing distance than the latter in their close-packed films, as has been confirmed by the following TEM data. As opposed to the CsPbBr_3_ NPLs with uneven size and shape distribution (Fig. S7a and Fig. 1), DBSA-CsPbBr_3_ NPLs show much more uniform size and shapes (Fig. S7b and Fig. 1), with two kinds of close-packing observed on the TEM images: NPLs either lying side-by-side or stacking face-to-face. In Fig. S7c, high-resolution TEM (HRTEM) image reveals cubic structure for DBSA-CsPbBr_3_ NPLs with 0.59 nm lattice spacing corresponding to the (100) plane^52^. From the statistical analysis of 100 particles, DBSA-CsPbBr_3_ NPLs have a thickness of ~1.9 nm, an edge length of ~6.2 nm, and an interparticle distance of ~2.3 nm between closely-packed NPLs (Fig. S7d–f). Considering the height of a [PbBr_6_]^4-^ octahedra in the perovskite structure being equal to 0.59 nm, the number of unit-cells in the DBSA-CsPbBr_3_ NPLs should be equal to 3.

### Crystallinity and structural stability

X-ray diffraction (XRD) measurements are conducted on drop-cast CsPbBr_3_ and DBSA-CsPbBr_3_ NPL films. Both kinds of NPLs exhibit strong diffraction peaks in small-angle XRD patterns (Fig. S8), which reflect the interplanar diffraction peaks of (002), (004), and (006) from the face-to-face stacking of the NPLs^53^. XRD patterns also confirm that the DBSA-CsPbBr_3_ NPL films are more orderly assembled as compared to CsPbBr_3_ NPL films, which results in a higher periodicity of the peaks at small angles. The diffraction peaks from CsPbBr_3_ and DBSA-CsPbBr_3_ NPL stacking appear at regular intervals of Δ2θ = 2.1° and 2.2°, respectively, corresponding to the spacing between face-to-face NPLs equal to 4.2 and 4.1 nm, respectively.

**Fig. 3:**
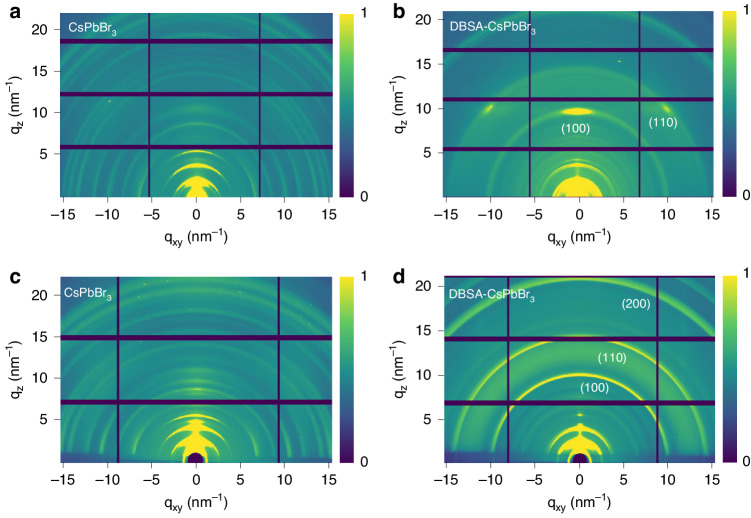
Structural characterization of CsPbBr_3_ and DBSA-CsPbBr_3_ NPLs. 2D GIWAXS patterns of **a** CsPbBr_3_ and **b** DBSA-CsPbBr_3_ NPLs, and 2D GIXRD patterns of **c** CsPbBr_3_ and **d** DBSA-CsPbBr_3_ NPLs

Combining grazing incidence wide-angle X-ray scattering (GIWAXS) and grazing incidence X-ray diffraction (GIXRD) measurements, we have further analyzed the structural details of CsPbBr_3_ and DBSA-CsPbBr_3_ NPL films. Spacings associated with the low-*q* peaks (namely diffraction peaks less than 10 nm^−1^) identify the periodic structure reflecting NPL placement^54^, while the high-*q* peaks reveal the crystal structure of CsPbBr_3_ perovskites. From GIWAXS patterns (Fig. 3a, b), equally spaced scattering rings of low-*q* values are recognized for both kinds of NPLs, indicating that they all possess periodic structures. As compared to the CsPbBr_3_ NPL films, DBSA-CsPbBr_3_ NPL films exhibit a preferential orientation with the appearance of strong, sharp, and discrete Bragg spots in the (110) and (100) planes. Such preferential orientation of DBSA-CsPbBr_3_ NPLs can be ascribed to their uniform morphology (Fig. 1), better monodispersity and reduced spatial hindrance when compared with the CsPbBr_3_ NPLs with various morphologies and various folding/bending forms of OLA-Br (DFT results shown in Figs. 2f and 2g), allowing for an orderly arrangement in films. In addition, the scattering intensity of the out-of-plane direction (*q*_z_) in DBSA-CsPbBr_3_ NPL films is much higher than that of the in-plane direction (*q*_xy_), suggesting that DBSA-CsPbBr_3_ NPLs are preferentially oriented parallel to the substrate (Fig. 3b). Data from the GIXRD measurements performed on the CsPbBr_3_ and DBSA-CsPbBr_3_ NPL films are presented in Figs. 3c and 3d, respectively. Similar to GIWAXS data, both films exhibit diffraction rings of relatively greater intensity along the *q*_z_ than the *q*_xy_ axis, indicating their preference for parallel orientation. The diffraction ring intensity for CsPbBr_3_ NPLs is strong only for the low-q peaks, while for DBSA-CsPbBr_3_ NPLs, it is also strong for the (100), (110), and (200) planes, suggesting much-improved crystallinity.

As enhanced crystal quality increases NPL resistance to external stimuli, we expect DBSA-CsPbBr_3_ NPLs to have higher stability. XRD patterns of the two samples are thus compared after storing them at ambient conditions for different period of time. Unlike films made of CsPbBr_3_ NPLs which decomposed completely within 10 days as judged from the XRD data (Fig. S9a), DBSA-CsPbBr_3_ NPL films maintain their original XRD patterns after 40 days of storage (Fig. S9b), indicating their enhanced stability. We also evaluate the PL stability of CsPbBr_3_ and DBSA-CsPbBr_3_ NPLs in films, freshly prepared and stored for different time periods under 40% relative humidity, as summarized in Fig. S9c. While both the PL peak position and PL bandwidth of the DBSA-CsPbBr_3_ NPL films remain almost unchanged after 15 days, the PL peak of CsPbBr_3_ NPL films red-shift by 13 nm to 472 nm, and a shoulder peak appears at 497 nm after 5 days of storage. As can be seen from the photographs of those films shown in Fig. S10, the CsPbBr_3_ NPL films lose their blue PL emission much faster than DBSA-CsPbBr_3_ NPL films.

Figure S11 provides the data related to evaluation of UV irradiation and thermal stability of CsPbBr_3_ and DBSA-CsPbBr_3_ NPLs. The PL intensities of CsPbBr_3_ and DBSA-CsPbBr_3_ NPL films decline to ~50% after continuous UV illumination for 4 min and 28 min (Fig. S11a), respectively, indicating a seven-fold increase of the half-life under UV illumination for the DBSA-CsPbBr_3_ NPLs. Figure S11b shows the thermal stability of the two kinds of NPL films, measured from 293 to 353 K. Upon reaching 353 K, CsPbBr_3_ and DBSA-CsPbBr_3_ NPL films lose 93% and 68% of their peak PL intensity, respectively. During the heating process, the PL spectra from DBSA-CsPbBr_3_ NPL film remain almost unchanged, while the PL spectra of CsPbBr_3_ NPL film shift from 463 to 490 nm as a result of the NPL aggregation. Consequently, the DBSA-CsPbBr_3_ NPL films show improved stability both towards UV irradiation and heat.

### Optical properties

**Fig. 4:**
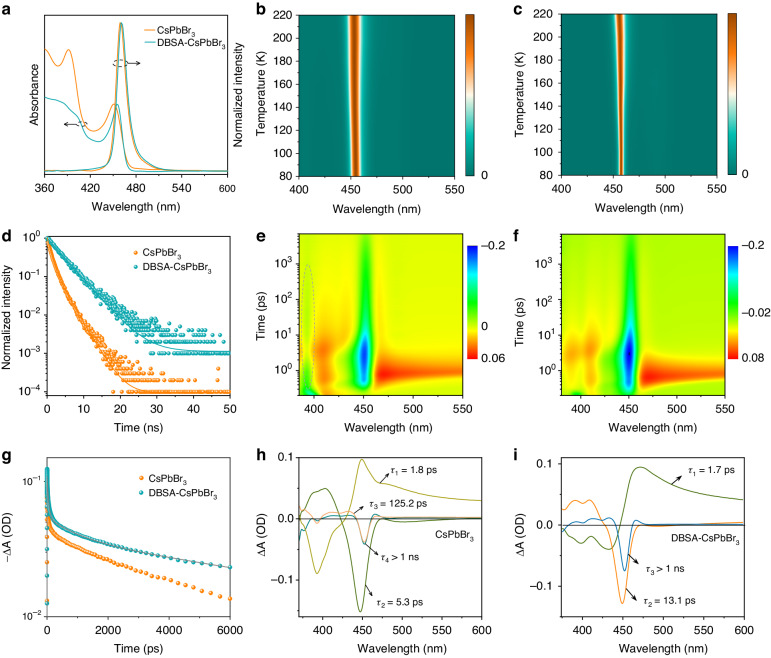
Optical properties of CsPbBr_3_ and DBSA-CsPbBr_3_ NPLs. **a** Steady-state absorption and PL spectra (365 nm excitation) of CsPbBr_3_ and DBSA-CsPbBr_3_ NPLs dissolved in toluene. Pseudocolor spectral map of temperature-dependent PL measured from 80 K to 220 K of **b** CsPbBr_3_ and **c** DBSA-CsPbBr_3_ NPLs. **d** Time-resolved PL decay curves of CsPbBr_3_ and DBSA-CsPbBr_3_ NPLs. TA plots of **e** CsPbBr_3_ and **f** DBSA-CsPbBr_3_ NPLs. **g** Comparison of the bleach recovery kinetics of CsPbBr_3_ and DBSA-CsPbBr_3_ NPLs measured at 455 nm. Characteristic decay-associated TA spectra for **h** CsPbBr_3_ and **i** DBSA-CsPbBr_3_ NPLs

Steady-state absorption and PL spectra of the two kinds of NPLs are shown in Fig. 4a. Because of the highly dynamic OLA-Br binding of CsPbBr_3_ NPLs, OLA and PbBr_2_ are generated upon decomposition of the NPL surface. Over time, OLA and PbBr_2_ concentration in solution would increase, causing molecular clusters to form again. Therefore, some molecular cluster absorption at 396 nm can still be detected in CsPbBr_3_ NPLs. Compared with CsPbBr_3_ NPLs, the absence of strong absorption at 396 nm in DBSA-CsPbBr_3_ NPLs indicates that the molecular clusters as impurities have been inhibited completely. Also in absorption, DBSA-CsPbBr_3_ NPLs exhibit a slight red shift of 4 nm of their first excitonic peak, as compared to CsPbBr_3_ NPLs, and an additional peak at 403 nm appears originating from higher-energy transition, which could only be observed in highly quantum-confined perovskite NPLs with a small Stokes shift and uniform morphology^55–57^. The PL maxima of CsPbBr_3_ and DBSA-CsPbBr_3_ NPLs are located around 460 nm, which corresponds to three vertically stacked unit-cells in NPLs. The Urbach tail is an indication of structural and electronic disorder in luminescent materials, and the Urbach energy (*E*_U_) is calculated to quantify the band edge broadening, according to the formulae *α* = *α*_0_exp[(*hν*−*E*_g_)/*E*_U_], where *α* is the absorption coefficient as a function of photon energy, *hν* is the photon energy, *E*_g_—the optical bandgap, and *α*_0_ is a constant. From Fig. S12, the *E*_U_ for CsPbBr_3_ NPLs is 27 meV, while for DBSA-CsPbBr_3_ NPLs it is much lower, namely 19 meV. This shows that DBSA-CsPbBr_3_ NPLs are less electronically disordered and have a lower trap density than CsPbBr_3_ NPLs^58^.

Figures 4b and c show pseudocolor maps of the temperature-dependent PL for CsPbBr_3_ and DBSA-CsPbBr_3_ NPLs, respectively, collected for the temperature range of 80–220 K. There is almost no shift in the PL peak for the both kinds of NPLs as the temperature increases. The DBSA-CsPbBr_3_ NPLs at 80 K have a bandwidth of 3.7 nm, which is much narrower than 8 nm of CsPbBr_3_ NPLs, again suggesting fewer electronic/structural disorder and scattering on defects (Fig. S13)^59–62^. When the temperature increases to 220 K, the PL bandwidth increases to 8 nm for DBSA-CsPbBr_3_ NPLs as compared to 11 nm for CsPbBr_3_ NPLs. Stronger change of the temperature-dependent PL bandwidth observed for the DBSA-CsPbBr_3_ NPLs indicates that excitonic recombination dominates at lower temperatures, demonstrating superior crystal quality of this sample^63^.

CsPbBr_3_ and DBSA-CsPbBr_3_ NPLs are further characterized using time-resolved PL (TRPL) spectroscopy, and their PL QYs are determined. Bi-exponential and the mono-exponential decays are used to fit the TRPL decay curves of CsPbBr_3_ NPLs and DBSA-CsPbBr_3_ NPLs, respectively (Fig. 4d). According to Table S2, the average PL lifetime (*τ*_ave_) increases from 1.8 ns in CsPbBr_3_ NPLs to 4.0 ns in DBSA-CsPbBr_3_ NPLs. Under 365 nm excitation, the PL QY of DBSA-CsPbBr_3_ NPLs reaches impressive 97% at room temperature in toluene solution, which is much higher than 10% in CsPbBr_3_ NPLs. Recombination rates for radiative (*K*_r_) and nonradiative (*K*_nr_) are calculated by using equations *K*_r_ = PLQY/*τ*_ave_ and *K*_nr_ = 1/*τ*_ave_ − *K*_r_, and the obtained values are summarized in Table 1. *K*_r_ of DBSA-CsPbBr_3_ NPLs increases 4-fold as compared to CsPbBr_3_ NPLs, while *K*_nr_ decreases 72-fold, which is due to the crystallinity and structural rigidity improvements brought out by using DBSA, which prevent the defect formation and thus eliminates nonradiative recombination.

**Table 1:** PL QYs, radiative recombination rates (*K*_r_), and nonradiative recombination rates (*K*_nr_) of CsPbBr_3_ and DBSA-CsPbBr_3_ NPLs

Sample	PL QY (%)	*K*_r_ (×10^7^ s^−1^)	*K*_nr_ (×10^7^ s^−1^)
CsPbBr_3_	10	5.6	50.0
DBSA-CsPbBr_3_	97	24.3	0.7

Femtosecond transient absorption (TA) spectra are used to examine the carrier dynamics of CsPbBr_3_ and DBSA-CsPbBr_3_ NPLs. Both kinds of NPLs exhibit photobleaching at 455 nm (Fig. 4e, f), which corresponds to the ground-state bleaching of the first excitonic transition. The photobleaching peak caused by molecular clusters is observed at 393 nm for CsPbBr_3_ NPLs, but not for DBSA-CsPbBr_3_ NPLs, in accordance with their absorption spectra (Fig. 4a). As shown in Fig. 4g, DBSA-CsPbBr_3_ NPLs have slower decay dynamics than CsPbBr_3_ NPLs, which is consistent with TRPL data discussed above. A more detailed picture of the carrier dynamic processes is revealed by decay-associated TA spectra, which are shown in Fig. 4h, i. The related time constants are extracted as follows: *τ*_1_ = 1.7 ps, *τ*_2_ = 13.1 ps, *τ*_3_ > 1 ns for DBSA-CsPbBr_3_ NPLs, and *τ*_1_ = 1.8 ps, *τ*_2_ = 125.2 ps, *τ*_3_ > 1 ns, *τ*_4_ = 5.3 ps for CsPbBr_3_ NPLs. Here, *τ*_1_, *τ*_2_, and *τ*_3_ account for intraband hot-exciton relaxation, exciton trapping in the bandgap trap states, and exciton recombination, respectively^7,64^. The former two processes are faster in DBSA-CsPbBr_3_ NPLs than in CsPbBr_3_ NPLs, suggesting that their enhanced crystal rigidity indeed lowers the defect density and suppresses electron-phonon coupling^65,66^. Besides, the *τ*_4_ component which is only present in CsPbBr_3_ NPLs represents the energy transfer from molecular clusters to CsPbBr_3_ NPLs; this component is absent in DBSA-CsPbBr_3_ NPLs.

### CsPbBr_3_ NPL-based LEDs

**Fig. 5:**
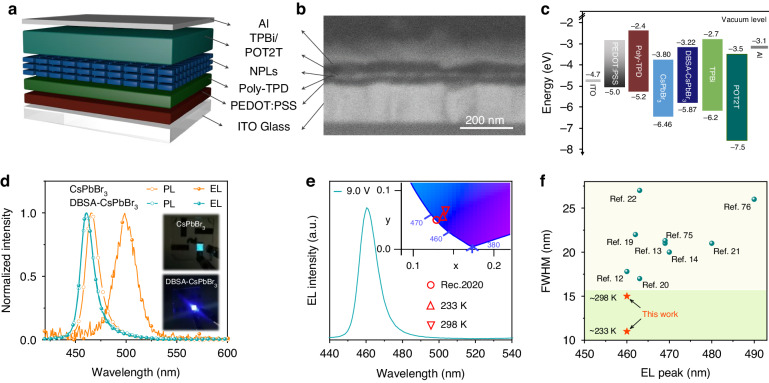
NPL-based LEDs. **a** Schematic illustration of the NPL-based LED structure, **b** its corresponding cross-sectional SEM image, and **c** its energy band diagram. **d** EL spectra of the LEDs based on CsPbBr_3_ and DBSA-CsPbBr_3_ NPLs (curves labeled with filled circles) and PL spectra of the respective NPL films (curves labeled with open circles). The insets show photographs of the LEDs based on CsPbBr_3_ and DBSA-CsPbBr_3_ NPLs operated under a 7 V bias. **e** EL spectrum of the DBSA-CsPbBr_3_ NPL-based LED operated at 233 K. The inset shows the CIE coordinates of the devices operated at 233 and 298 K, which are compared with the Rec. 2020 CIE coordinates for blue light applied for ultra-high-definition displays. **f** Comparison of the EL bandwidth of the reported blue LEDs based on CsPbBr_3_ and (CsPbCl_*x*_Br_1-*x*_)_3_ NPLs and nanocrystals, and the values obtained for our DBSA-CsPbBr_3_ NPL-based LED at 233 and 298 K^12–14,19–22,75,76^

For the demonstration of the benefits of enhanced structural rigidity, both kinds of perovskite NPLs have been used as emitter layers in LEDs, in order to compare their EL performance. Figure 5a illustrates the LED architecture, which includes indium tin oxide (ITO)/poly-(ethylene dioxythiophene):polystyrene sulfonate (PEDOT: PSS)/poly (N,N9-bis(4-butylphenyl)-N,N9-bis(phenyl)-benzidine) (Poly-TPD)/polyethyleneimine (PEI)/NPLs/1,3,5-Tris(1-phenyl-1H-benzimidazol-2-yl)benzene (TPBi)/2,4,6-Tris[3-(diphenylphosphinyl)phenyl]-1,3,5-triazine (POT2T)/Al. Here, the ITO substrate serves as the anode, a bilayer of PEDOT: PSS/Poly-TPD with a thickness of ~30 nm—as the hole transport layer (HTL), the layer of CsPbBr_3_ NPLs (~12 nm)—as the emitting layer, a bilayer of TPBi/POT2T (~40 nm)—as the electron transport layer (ETL), and Al layer (~100 nm)—as the cathode. A scanning electron microscope (SEM) in Fig. 5b visualizes the device structure and the thicknesses of each functional layer.

Ultraviolet photoelectron spectroscopy (UPS) is used to determine the valence band maximum (VBM) and the Fermi energy level (*E*_F_) for both kinds of perovskite NPLs. For the DBSA-CsPbBr_3_ NPLs, the VBM upshifts from −6.46 to −5.87 eV as compared to the reference CsPbBr_3_ NPLs, while the *E*_F_ downshifts from −3.90 to −3.47 eV (Fig. S14a). Consequently, emitting layers of DBSA-CsPbBr_3_ NPLs have higher hole concertation than of the CsPbBr_3_ NPLs resulting in a faster hole transport recorded from the “hole-only” devices of Fig. S15. Moreover, for the films made of DBSA-CsPbBr_3_ NPLs, the hole injection barrier decreases from 1.26 to 0.67 eV as compared to the films of CsPbBr_3_ NPLs (Fig. 5c). From the Tauc plots of the perovskite films on quartz substrates provided in Fig. S14b, the bandgap values of CsPbBr_3_ and DBSA-CsPbBr_3_ NPLs are determined as 2.68 eV and 2.67 eV, respectively. The upward moving conduction band minimum (CBM) reduces the energy level difference between the ETL and the emitting layer by 0.58 eV in the LED based on DBSA-CsPbBr_3_ NPLs (Fig. 5c), which should result in a more reliable and stable LEDs. Space-charge limited-current measurements are performed on the hole-only and electron-only devices (with the device structure provided in Fig. S14 caption) to further demonstrate the advantages of the surface engineering using DBSA. When operated at low voltages, the current density in the hole-only devices based on DBSA-CsPbBr_3_ NPLs is higher, while in their electron-only devices it is lower, which suggests an improved charge balance in LEDs as compared to respective devices based on CsPbBr_3_ NPLs. Meanwhile, the current density-voltage curve for the CsPbBr_3_ NPL hole-only device increases sharply under high voltage, which is a signature of the material degradation. Hole and electron trap-filled limit voltages (*V*_TFL_) of the devices based on DBSA-CsPbBr_3_ NPLs (0.91 and 2.02 V) are both lower than those based on CsPbBr_3_ NPLs (1.15 and 2.40 V). Moreover, DBSA-CsPbBr_3_ NPLs show lower trap density than CsPbBr_3_ NPLs (Table S3), which is in a good agreement with removal of the surface defects by the treatment with DBSA.

From the current density-voltage-luminance (*J-V-L*) curves shown in Fig. S16a, DBSA-CsPbBr_3_ NPL-based LEDs exhibit a lower turn-on voltage (3.0 V), as well as higher current densities and brightness than the LED based on CsPbBr_3_ NPLs. While the CsPbBr_3_ NPL-based LEDs show external quantum efficiency (EQE) of only 0.08% and the brightness of 55 cd m^−2^, the DBSA-CsPbBr_3_ NPL-based LEDs with the respective values of 1.60% and 591 cd m^−2^ are 20 times more efficient and more than 10 times brighter (Fig. S16b). Importantly, the averaged EQE value recorded for 30 DBSA-CsPbBr_3_ NPL-based LEDs is 1.22% (Fig. S17), demonstrating high reproducibility. In terms of the device stability, the EL spectrum red-shifts from 465 nm (PL of the film) to 500 nm and the bandwidth broadens to 24 nm from 16 nm in LEDs using CsPbBr_3_ NPLs (Fig. 5d), so that the emission color of the operating device changes from blue to green (inset in Fig. 5d), which is due to the poor thermal stability (Fig. S11b, c). Because the electron transport layer TPBi is directly deposited on top of the NPL film via thermal evaporation, high temperature vapor facilities the detachment of ligands, leading to the CsPbBr_3_ NPL aggregation. In contrast, the DBSA-CsPbBr_3_ NPL-based LEDs show spectrally symmetrical, homogeneous, and bright color-saturated blue emission peaked at 460 nm, with an excellent color stability as demonstrated in Fig. 5d, which is due to the stable NPL surface resulting from strongly bound DBSA. Furthermore, benefitting from the superior structural stability of the DBSA-CsPbBr_3_ NPLs, when the voltage increases from 4.5 to 6.5 V, the EL peak and bandwidth of the color-saturated blue LEDs remain almost unchanged (Fig. S18), indicating that the color purity and CIE coordinates are not affected. Thus, the device’s EL meets the standard for high-definition displays throughout their operation. By comparison with the reported blue LEDs based on CsPbBr_3_ and CsPb(Cl_*x*_Br_1-*x*_)_3_ NPLs and nanocrystals provided in Fig. 5f, DBSA-CsPbBr_3_ NPL LEDs have the narrowest EL bandwidth of 15 nm at 298 K and 11 nm at 233 K. Since DBSA-CsPbBr_3_ NPLs with superior crystal quality possess fewer electronic defects or structural disorders, phonon scattering is suppressed at low temperatures, so that the excitonic recombination characterized by a narrow emission bandwidth dominates. The EL spectra at 298 K and 233 K corresponds to CIE coordinates of (0.141, 0.062) and (0.138, 0.050), resulting in CIE *y*-coordinate values of 0.062 and 0.050 below 0.15 and (*x* + *y*)-values of 0.203 and 0.188 below 0.30, which fully meeting Rec. 2020 standards of (0.131, 0.046) (Fig. 5e), illustrating their great potential as emitters for the high-definition displays.

## Discussion

In summary, surface engineering with DBSA ligands significantly enhances the crystal integrity and structural rigidity of CsPbBr_3_ NPLs, enabling a color-saturated, extremely narrow blue emission with nearly unity absolute PL QY. As determined by DFT calculations and supported by experimental data, covalent bonds are formed between the surface Pb^2+^ cations and DBSA with a binding energy of 5.16 eV, which is much higher than the energy of the ionic bonds between OLA-Br and Pb^2+^ (1.21 eV). The interaction between DBSA and perovskite NPL results in an ordered and stable perovskite surface, and thus yields CsPbBr_3_ NPLs with uniform shapes and morphology. Moreover, the covalent bonding with DBSA inhibits the phase transition and decomposition of perovskite phase, enabling CsPbBr_3_ NPLs with excellent structural and optical stability. The PL bandwidth of the color-saturated blue emission of the DBSA-CsPbBr_3_ NPLs measured at 80 K peaks at 457 nm and reaches an extremely narrow value of 3.7 nm. In comparison with other blue perovskite LEDs, the LEDs based on DBSA-CsPbBr_3_ NPLs show the narrowest EL bandwidth of 15 nm which corresponds to CIE coordinates of (0.141, 0.062), indicating their significant potential as emitters for ultra-high-definition displays meeting Rec. 2020 standards. DBSA-CsPbBr_3_ NPLs have demonstrated significantly improved optical performance and stability, which brings blue emitting perovskites closer to commercialization. The DBSA-assisted perovskite nanomaterials can thus be applied in a variety of applications requiring excellent optical performance, including lighting, signaling, and optical communications.

## Materials and methods

### Materials

Cesium bromide (CsBr, 99.999%), lead bromide (PbBr_2_, 99.999%), oleic acid (OA, 90%), dimethyl sulfoxide (DMSO, 99.5%), chlorobenzene (CB, 99.5%), and polyethyleneimine (PEI) were obtained from Sigma-Aldrich. Oleylamine (OLA, 80-90%), methyl acetate (99%), and N-butanol (99.8%) were obtained from Aladdin. Hydrobromic acid (40 wt% in H_2_O) was purchased from Tianjin Chemical Industry. 4-Dodecylbenzene sulfonic acid (DBSA, 95%) was purchased from Shanghai Acmec Biochemical Co., Ltd. Poly(3,4-ethylenedioxythiophene) polystyrene sulfonate (PEDOT:PSS), poly(4-butylphenyl-diphenyl-amine) (Poly-TPD), 1,3,5-tris(1-phenyl-1H-benzimidazol-2-yl)benzene (TPBi), and 2,4,6-Tris[3-(diphenylphosphinyl)phenyl]-1,3,5-triazine (POT2T) were purchased from Xi’an Polymer Light Technology Corp. Hexane, ethanol, dichloromethane, isopropyl alcohol, toluene, and acetone were obtained from Sinopharm Chemical Reagent Co., Ltd.

### Synthesis of CsPbBr_3_ and DBSA-CsPbBr_3_ NPLs

The syntheses were conducted under open-air conditions, following the LARP approach. In the first step, 0.1 mmol CsBr was dissolved in 1 mL of 40% HBr, and 1 mmol PbBr_2_ was dissolved in 2 mL DMSO. To produce CsPbBr_3_ NPLs, 100 μL PbBr_2_ precursor was rapidly injected into a 6 mL solution which contained 0.5 mL OA, 0.5 mL OLA, and 5 mL toluene, under continuous stirring. Then, 0.1 mL CsBr precursor was rapidly injected, and after appearance of a turbid white substance, 0.4 mL N-butanol was introduced. Subsequently, centrifugation at 8000 rpm was immediately performed and the supernatant containing CsPbBr_3_ NPLs was collected. After annealing the supernatant at 65 °C for 1 min, 2-fold volume excess of methyl acetate was added for purification under centrifugation at 10,000 rpm for 5 min. Finally, the precipitate was collected and redispersed in 1 mL toluene. For the synthesis of DBSA-CsPbBr_3_ NPLs, 100 mg of DBSA ligand was introduced into the mixture of 0.5 mL OA, 0.5 mL OLA, and 5 mL toluene, before injection of the PbBr_2_ precursor. Other steps were the same as described above.

### LED fabrication

Glass substrates with patterned ITO-coating were ultrasonically washed in sequence with deionized water, ethanol, dichloromethane, and isopropyl alcohol for 20 min, and then further cleaned with an oxygen-plasma for 15 min. A PEDOT:PSS solution was spin-coated onto ITO substrates at 4000 rpm for 40 s, followed by annealing at 150 °C for 15 min in ambient conditions. The substrate was transferred into a N_2_-filled glovebox, and poly-TPD solution (5 mg mL^–1^, CB) was spin-coated on top of PEDOT:PSS at 4000 rpm for 40 s, followed by annealing at 150 °C for 15 min. Subsequently, PEI solution (0.15 mg mL^−1^ in isopropyl alcohol) was spin-coated onto the poly-TPD layer at 2000 rpm for 40 s. A 12 nm NPL film (consisting of either CsPbBr_3_ or DBSA-CsPbBr_3_ NPLs) was deposited on PEI by spin-coating the NPL solution at 2000 rpm for 40 s. Finally, 5 nm TPBi, 35 nm POT2T, and 100 nm Al films were deposited by thermal evaporation in a vacuum deposition chamber (∼5 × 10^–4^ Pa pressure). A luminous area of 0.04 cm^2^ was defined as the overlap between ITO and Al electrodes.

### Characterization

FTIR spectra were taken on an IFS-66V/S FTIR spectrophotometer. TEM and HRTEM images were obtained measured on a JEM-2100F transmission electron microscope. XPS measurements were performed on an ESCALAB250 spectrometer. UV-vis absorption spectra were measured on a UV-2600 (Shimadzu) spectrophotometer, and PL spectra on a FLS920P fluorescence spectrometer. Absolute PL QYs were measured on an Edinburgh FLS820-s spectrometer with a calibrated integrated sphere. 1H NMR spectra were obtained at room temperature on a Zhongke Niujin AS400 (400 MHz, 1H) instrument. TRPL measurements were conducted on a time-dependent single photon counting system based on the FLS920P Edinburgh spectrometer with an excitation wavelength of 365 nm. TA spectra were measured using a femtosecond transient absorption pump-probe spectrometer (Ultrafast Systems LLC) with a pump wavelength of 365 nm. XRD patterns were obtained on a Bruker D8 Advance X diffractometer with Cu K_α_ Source (λ = 1.5406 Å). SEM images were collected on a Hitachi SU8000 SEM (Hitachi Limited, Tokyo, Japan) under 5 kV acceleration voltage. UPS measurements were performed on a PREVAC system. LED performance was evaluated using the commercially available system (SHENZHEN PYNECT SCIENCE AND TECHNOLOGY Co, Ltd.), with current density-voltage characteristics been recorded on a Keithley 2400 source meter, and light-output measurements on a fiber integration sphere coupled with a QE Pro spectrometer.

### Computational methods

The density functional theory (DFT) method as implemented in the Vienna ab initio simulation package (VASP)^67^ was adopted. Electron–ion interactions were described using projector-augmented wave pseudopotentials^68^. The generalized gradient approximation^69^ of the Perdew, Burke, and Ernzernhof functional (PBE)^70^ was employed. A plane-wave kinetic-energy cutoff of 600 eV and a *k*-spacing of 0.18 Å^−1^ in reciprocal space were adopted to achieve reliable results. The Van der Waals (vdW) interaction was considered by adopting optB86b-vdW functional^71^, which has been used in similar systems in previous studies^72^. The dipole correction was involved to cancel the artificial field between spurious images due to periodic boundary conditions. All atomistic structures were visualized using VESTA software^73^. The adsorption energy (*E*_ad_) was calculated using the following formula^74^: *E*_ad_ = *E*_ad_model_ − *E*_separate_model_, where *E*_ad_model_ is the total energy of the adsorption model, and *E*_separate_model_ is the total energy of the model where substrate and ligand were separated by the vacuum with a thickness of 15 Å to eliminate the interaction between them.

## Date availability

The data that support the plots within this paper and the other findings of this study are available from the corresponding authors upon reasonable request.

### Supplementary information

Supporting information
