# Supplementary material for: Enhancing crystal integrity and structural rigidity of CsPbBr3 nanoplatelets to achieve a narrow color-saturated blue emission
Source: Light Sci Appl. 2024 May 11;13:111. doi: 10.1038/s41377-024-01441-1 (PMC11088658; doi:10.1038/s41377-024-01441-1)
Supplement: Supplementary file 1 — Supporting information [file 41377_2024_1441_MOESM1_ESM.docx]

**Supporting Information**

*for*

**Enhancing crystal integrity and structural rigidity of CsPbBr_3_ nanoplatelets to achieve narrow color-saturated blue emission**

*Qianqian Huang^1, #^, Wenxu Yin^1, #^, Bo Gao^1^, Qingsen Zeng^2^, Dong Yao^2^, Hao Zhang^2^, Yinghe Zhao^3^, Weijia Zheng^4, *^, Jiaqi Zhang^1^, Xuyong Yang^5^,* *Xiaoyu Zhang^1, *^, and Andrey L. Rogach^6*^*

^1^ Key Laboratory of Automobile Materials MOE, School of Materials Science & Engineering, and Jilin Provincial International Cooperation Key Laboratory of High-Efficiency Clean Energy Materials, Jilin University, Changchun 130012, P.R. China

^2^ State Key Laboratory of Supramolecular Structure and Materials, College of Chemistry, Jilin University, Changchun 130012, P.R. China

^3^ State Key Laboratory of Materials Processing and Die & Mould Technology, School of Materials Science and Engineering, Huazhong University of Science and Technology, Wuhan, Hubei 430074, China

^4^ Department of Chemistry, University of Victoria, Victoria, BC V8P 5C2, Canada

^5^ Key Laboratory of Advanced Display and System Applications of Ministry of Education, Shanghai University, Shanghai 200072, P.R. China

^6^ Department of Materials Science and Engineering, and Centre for Functional Photonics (CFP), City University of Hong Kong, Hong Kong S.A.R 999077, P.R. China

^#^ These authors contributed equally to this work.

*^*^* Corresponding authors: Weijia Zheng (E-mail: [alex610@foxmail.com](mailto:alex610@foxmail.com))

Xiaoyu Zhang (E-mail: [zhangxiaoyu@jlu.edu.cn](mailto:zhangxiaoyu@jlu.edu.cn))

Andrey L. Rogach (Email: andrey.rogach@cityu.edu.hk)

**Supplementary Figures**


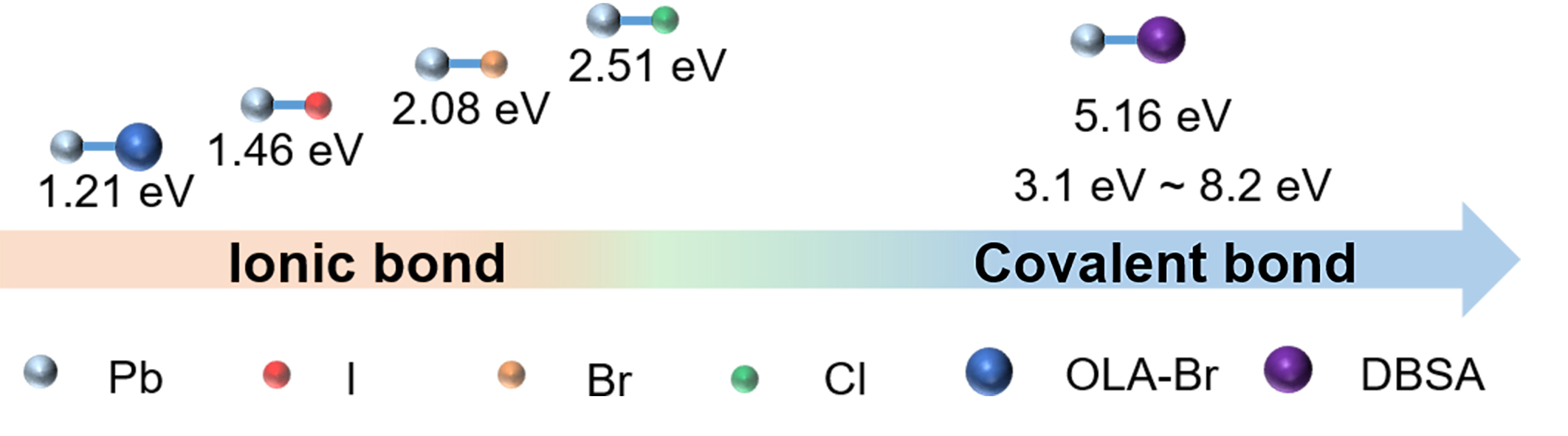


**Fig. S1:** Binding energy of Pb-X (X = Cl, Br, and I) and DBSA-Pb bonds. The adsorption energy between Pb^2+^ and deprotonated DBSA is estimated by DFT calculations, while the other values are taken from the book *Experimental Data of Chemical Bond Energies*.


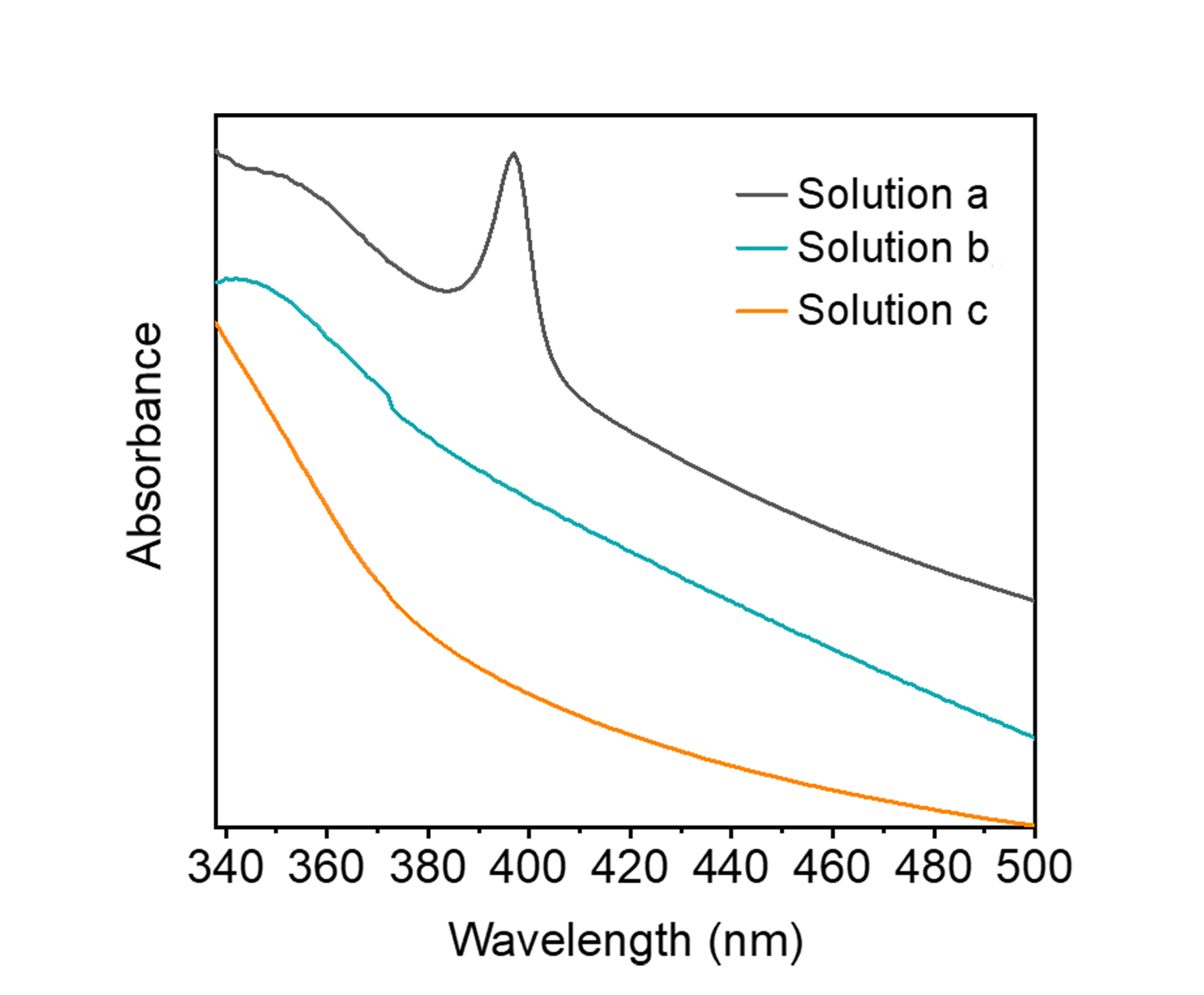


**Fig. S2:** Absorption spectra of 100 μL PbBr_2_ precursor with 500 μL OLA (Solution a), 100 mg DBSA (Solution b), and 500 μL OLA together with 100 mg DBSA (Solution c), all in toluene.


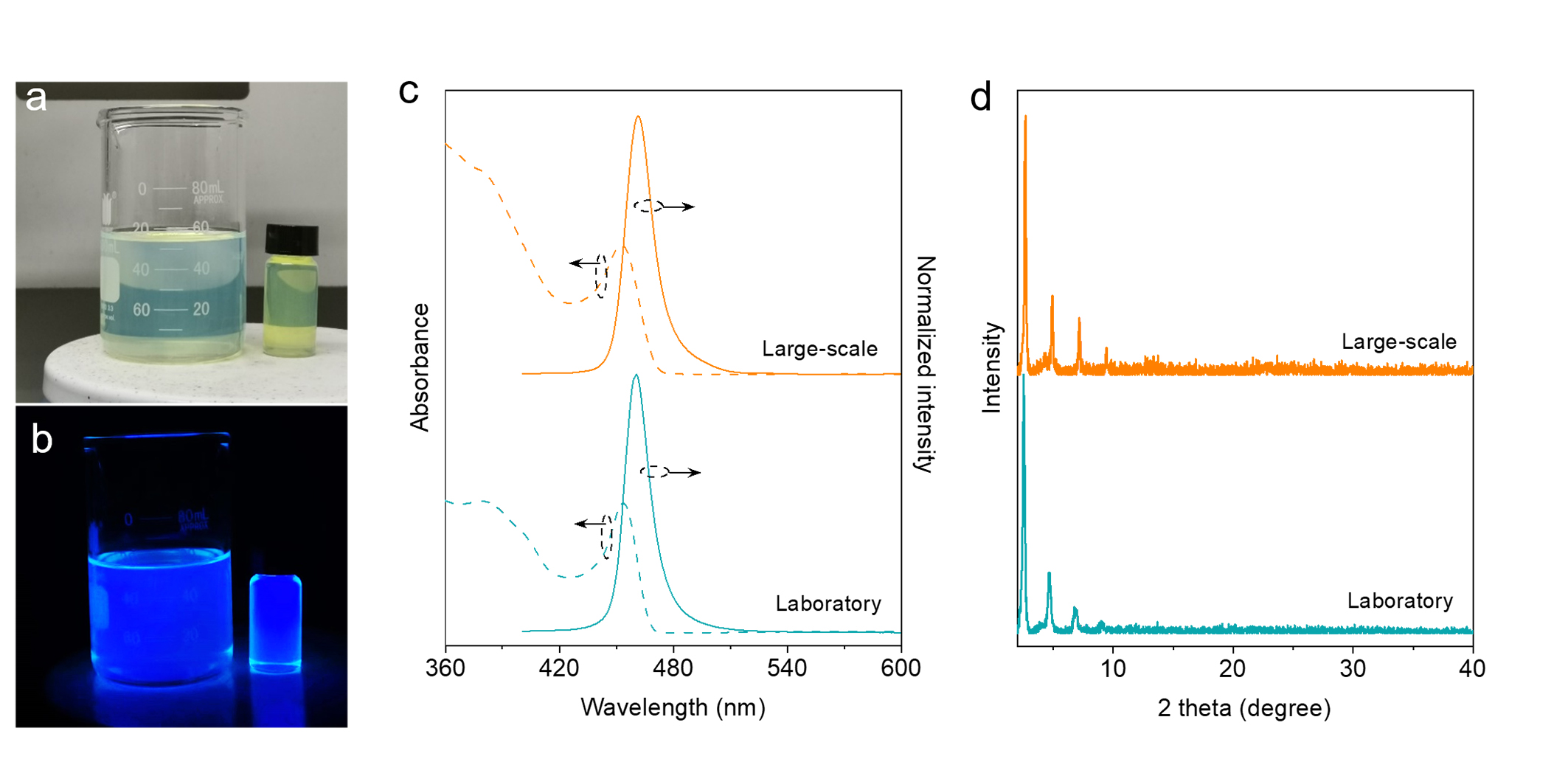


**Fig. S3:** Photographs of CsPbBr_3_ NPLs prepared by large-scale (ten-times large volume of 50-and-5 mL) and lab-level synthesis, taken under (a) daylight and (b) 365 nm excitation. (c) Absorption/PL spectra and (d) XRD patterns of CsPbBr_3_ NPLs originated from the large-scale and lab-level synthesis.


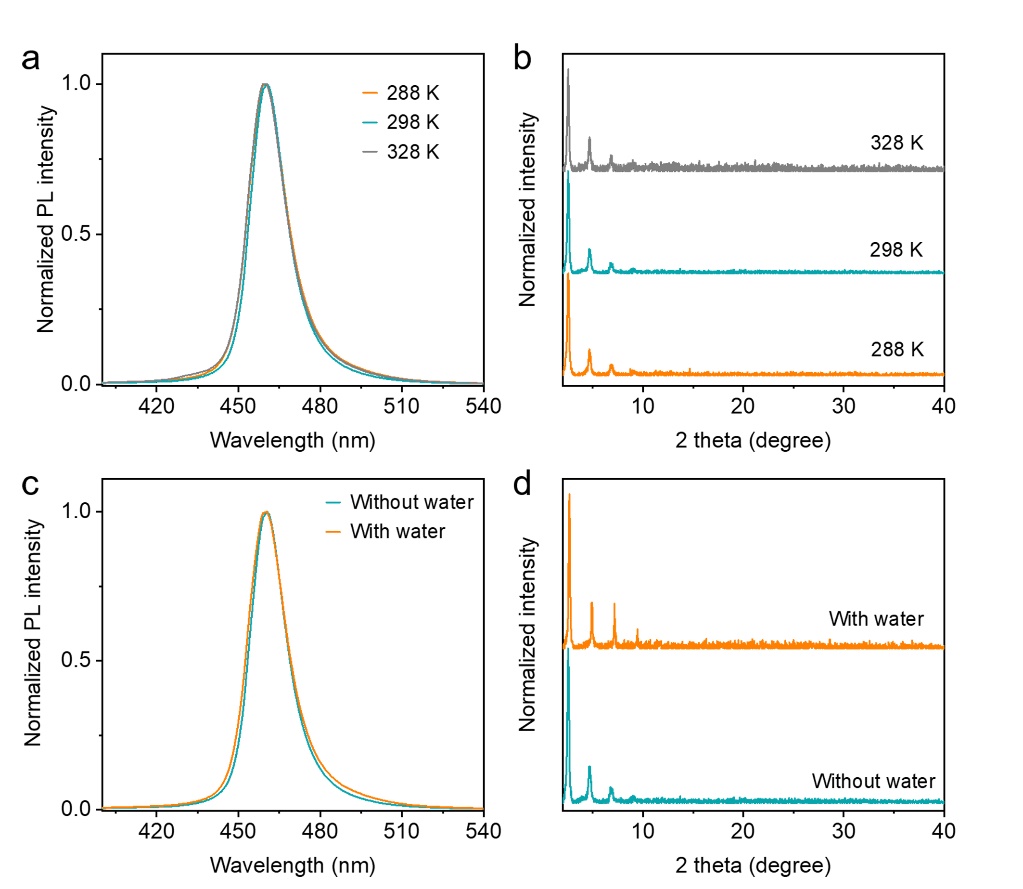


**Fig. S4:** (a, c) Normalized PL spectra and (b, d) XRD patterns of DBSA-CsPbBr_3_ NPLs obtained (a, b) at different temperatures and (c, d) with/without water additives.


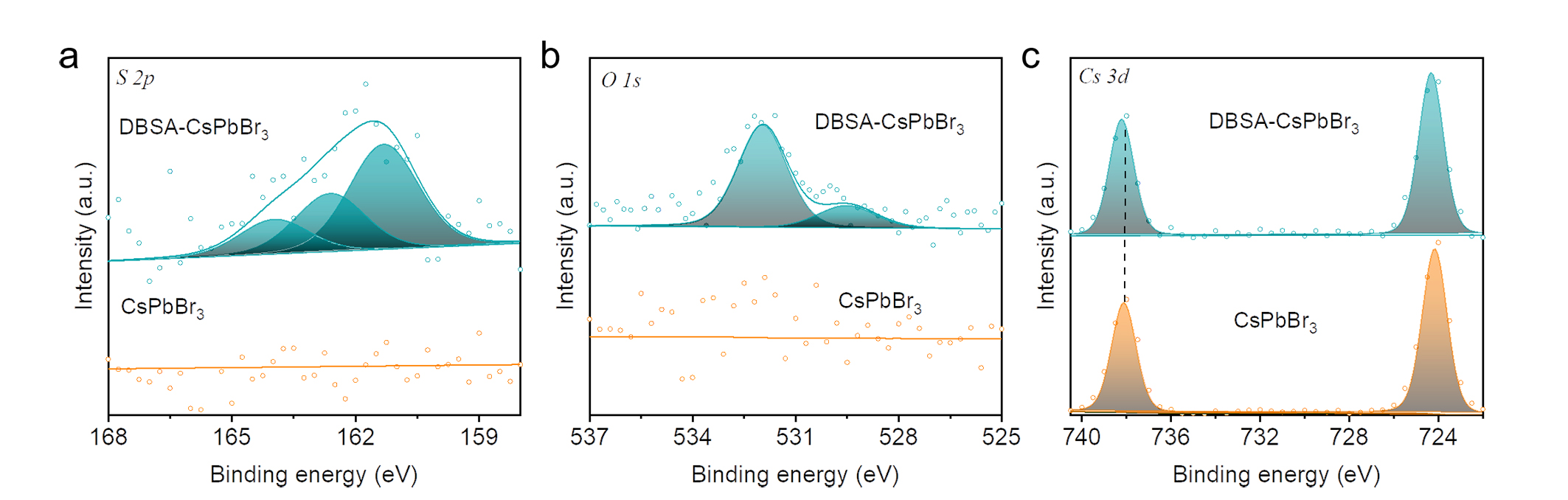


**Fig. S5:** High-resolution XPS spectra of (a) S *2p*, (b) O *1s*, and (c) Cs *3d* measured for CsPbBr_3_ and DBSA-CsPbBr_3_ NPLs.


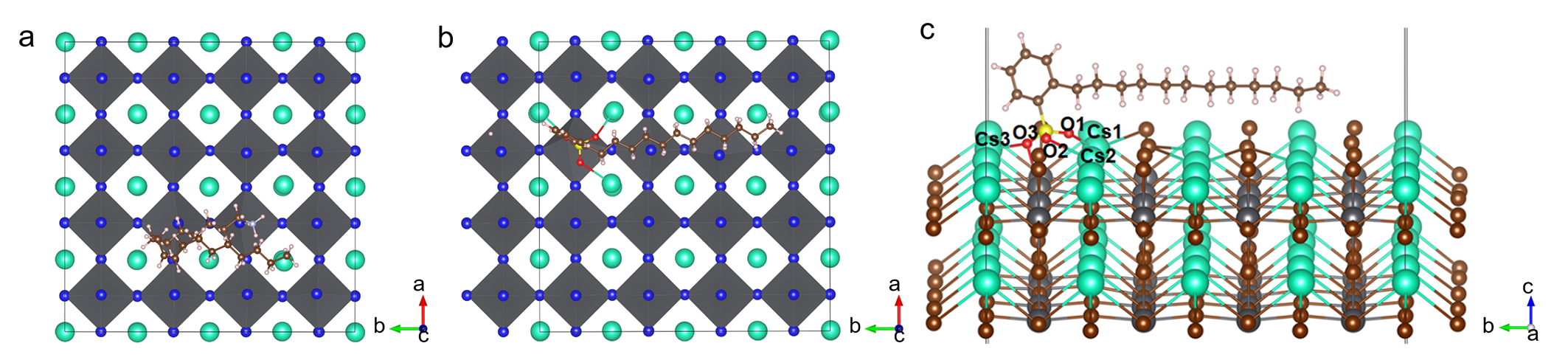


**Fig. S6:** A model of the adsorption between the surface of CsPbBr_3_ NPLs and (a) OLA-Br and (b) DBSA ligands. (c) Illustration of multiple coordination between DBSA and NPL surface, assuming one Pb^2+^ and three Cs^+^. The gain and loss of the electrons, and the bond lengths between Cs and O atoms derived from this model are summarized in Table S1.


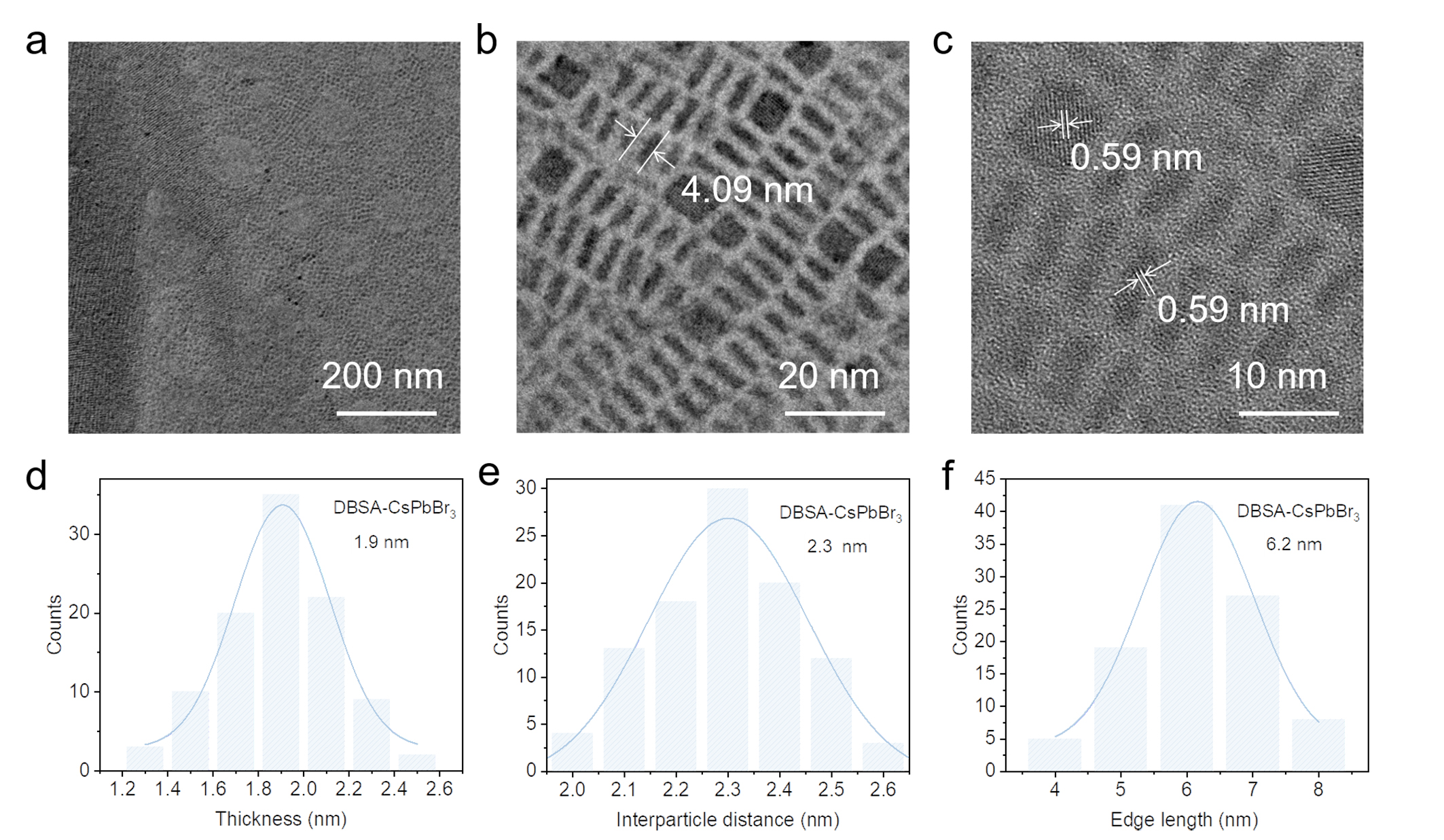


**Fig. S7:** TEM images of (a) CsPbBr_3_ and (b) DBSA-CsPbBr_3_ NPLs. The spacing of 4.09 nm in (b) is the face-to-face distance between NPLs. (c) HRTEM image of DBSA-CsPbBr_3_ NPLs. The spacing of 0.59 nm corresponds to the distance along the (100) lattice plane. (d) Estimated thickness distribution of DBSA-CsPbBr_3_ NPLs, (e) inter-NPL distance between DBSA-CsPbBr_3_ NPLs, and (f) edge length distribution of DBSA-CsPbBr_3_ NPLs.


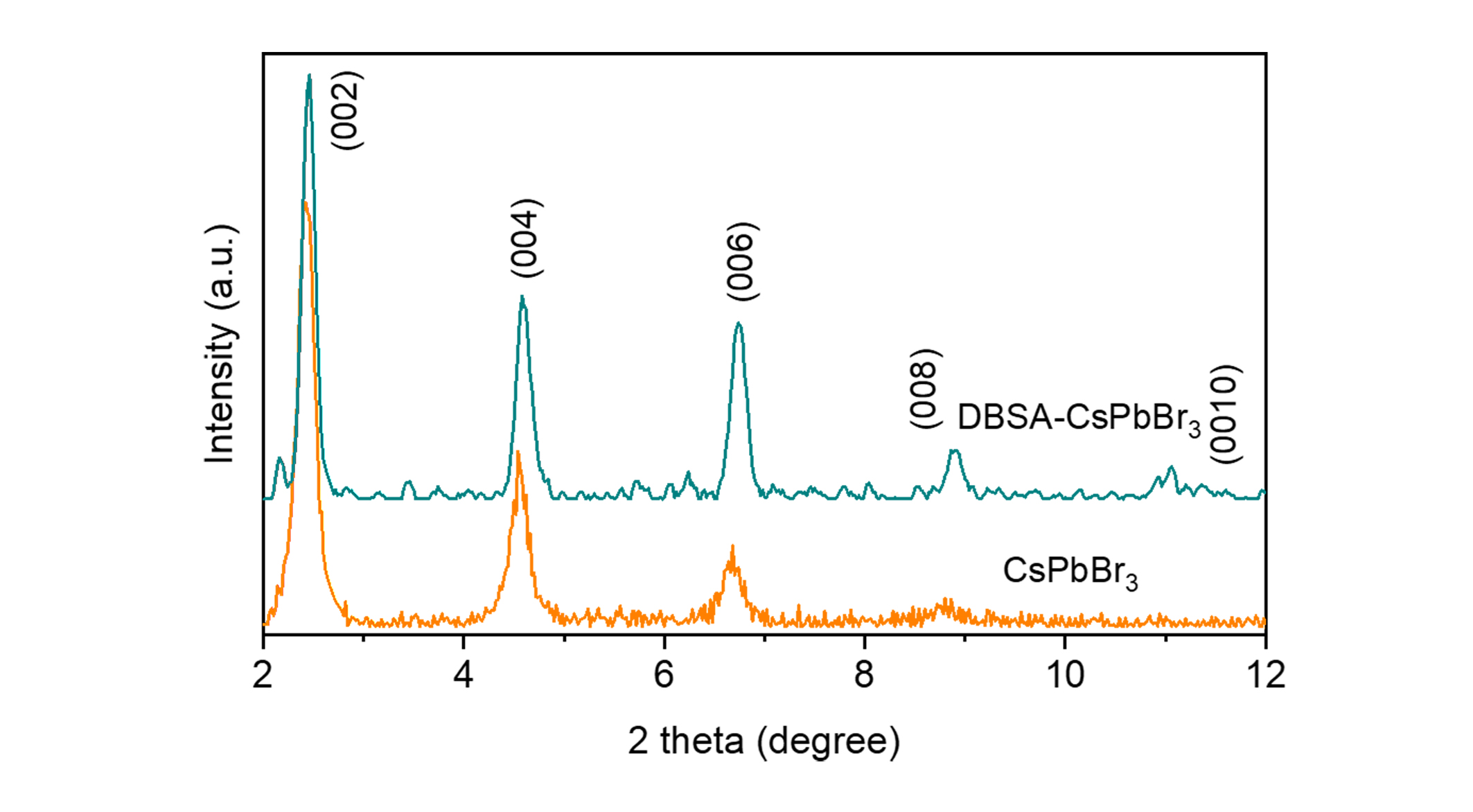


**Fig. S8:** Small-angle XRD patterns of closely-packed CsPbBr_3_ and DBSA-CsPbBr_3_ NPLs.


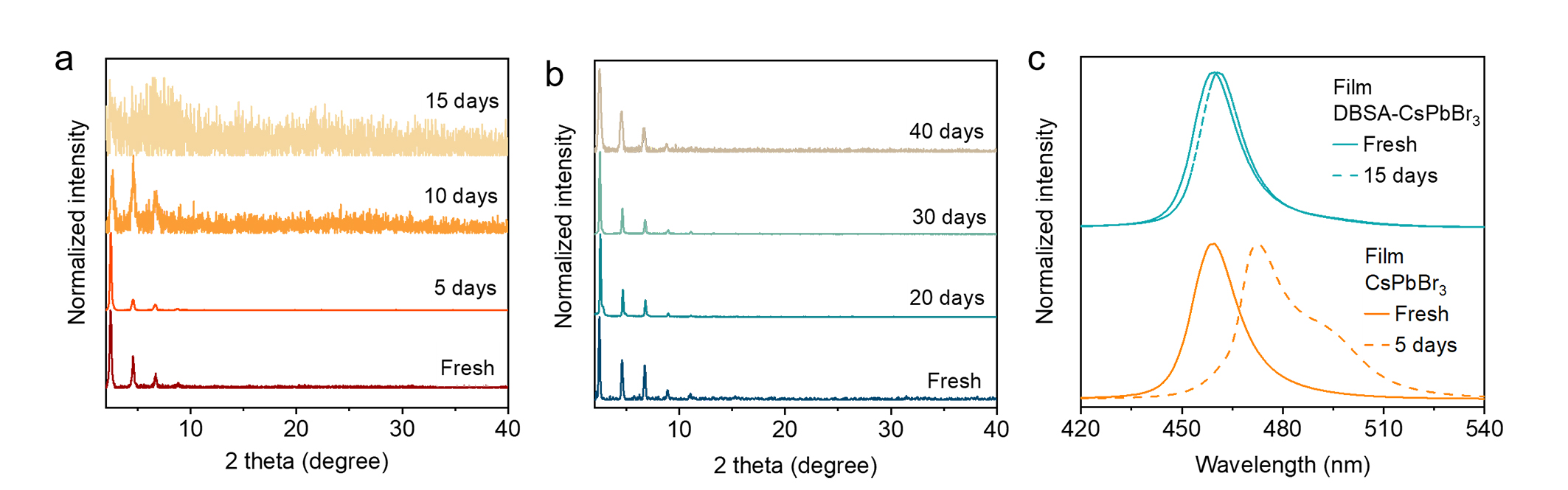


**Fig. S9:** XRD patterns of thin films made from (a) CsPbBr_3_ NPLs and (b) DBSA-CsPbBr_3_ NPLs. (c) PL stability of CsPbBr_3_ and DBSA-CsPbBr_3_ NPL films, freshly made and stored for different time under 40% relative humidity.


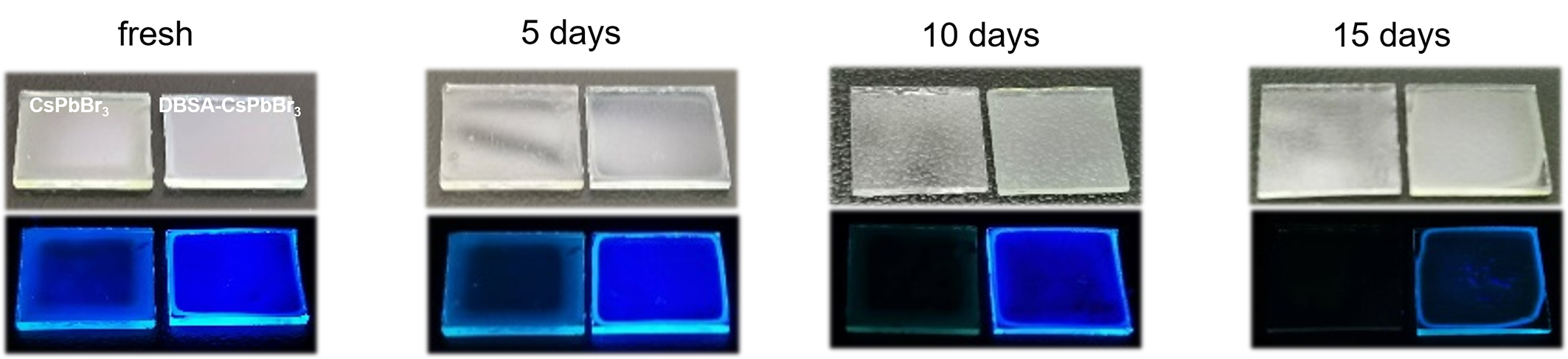


**Fig. S10:** Photographs of CsPbBr_3_ (always on the left) and DBSA-CsPbBr_3_ (always on the right) NPLs in their films exposed to 40% relative humidity, which were taken under day light (upper rows) and under 365 nm UV excitation (bottom rows).


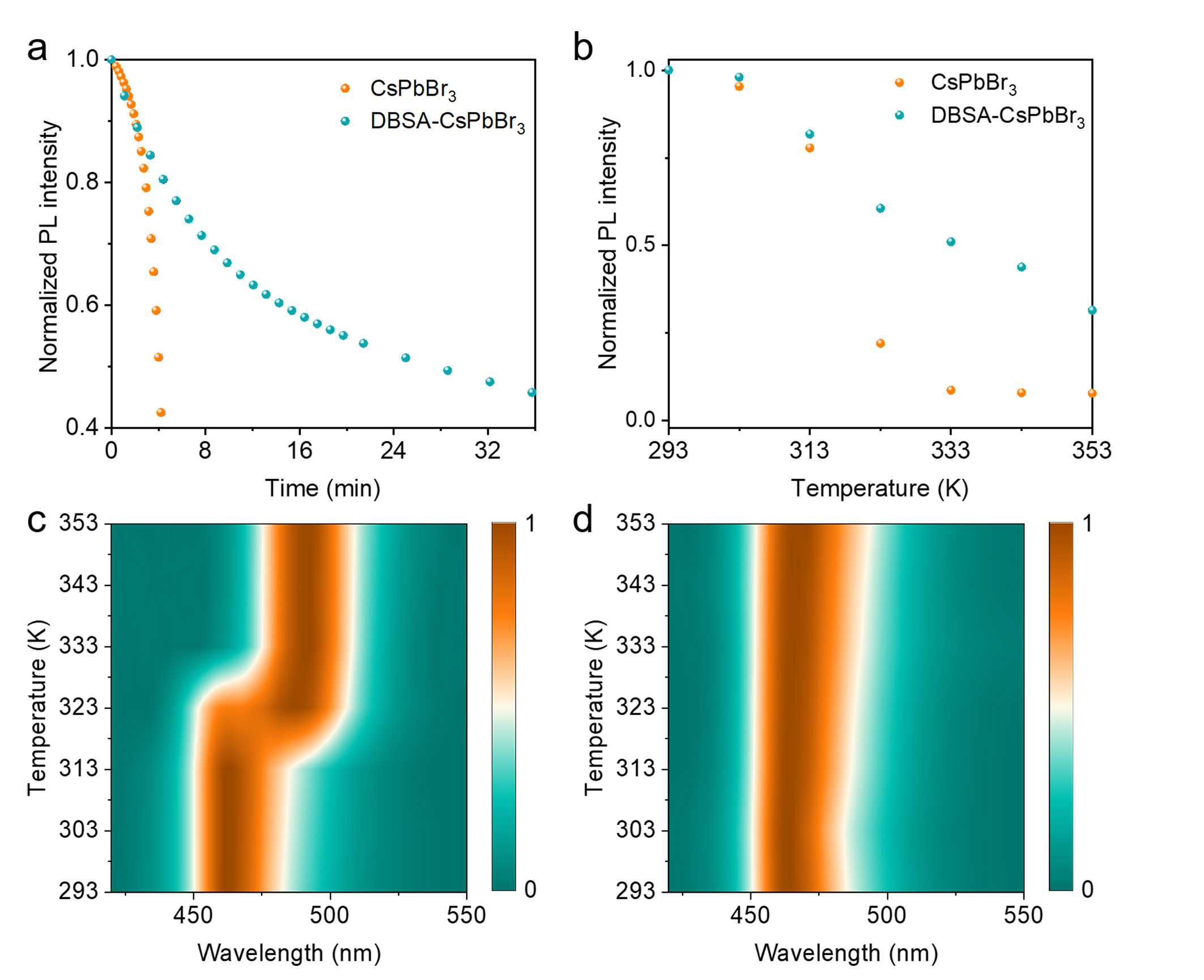


**Fig. S11:** (a) Stability under UV irradiation and (b) thermal stability of CsPbBr_3_ and DBSA-CsPbBr_3_ NPL films. Both films were exposed to continuous 365 nm illumination. Pseudocolor PL spectral maps measured from 293 K to 353 K of (c) CsPbBr_3_ and (d) DBSA-CsPbBr_3_ NPL films.


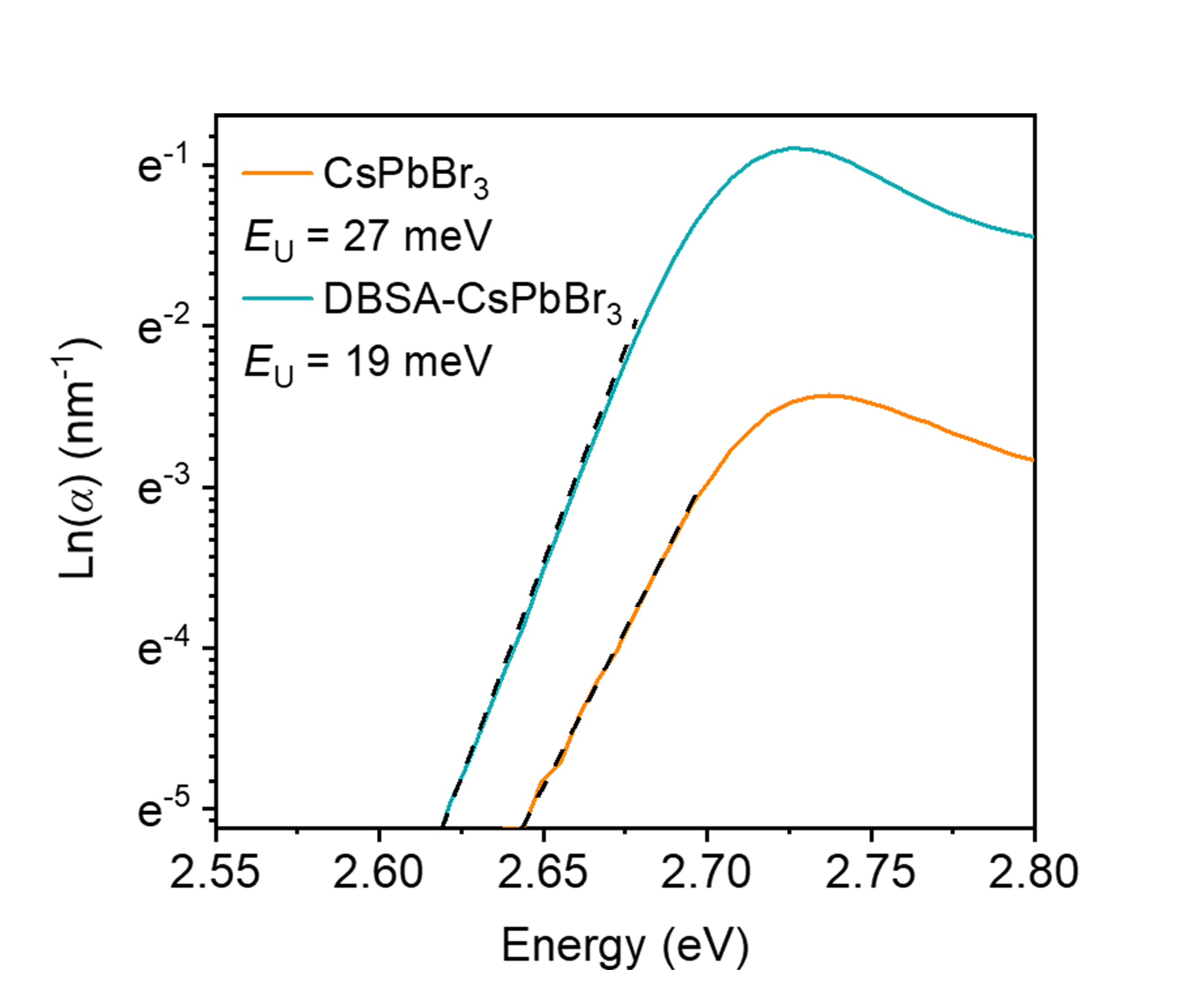


**Fig. S12:** Absorption coefficient as a function of photon energy for determination of the Urbach energy (*E*_U_) for CsPbBr_3_ and DBSA-CsPbBr_3_ NPLs.


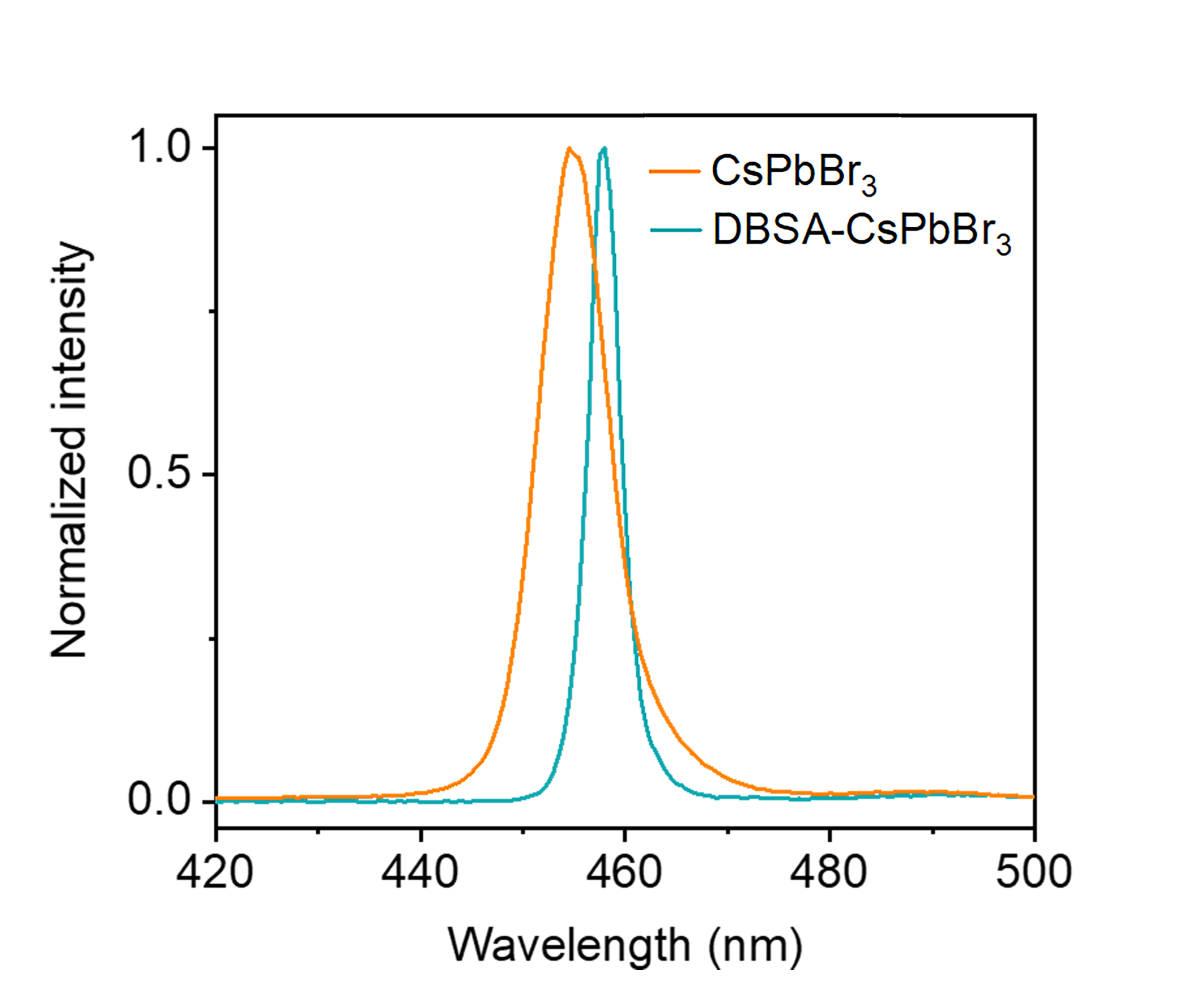


**Fig. S13:** PL spectra of CsPbBr_3_ and DBSA-CsPbBr_3_ NPLs, measured at 80 K.


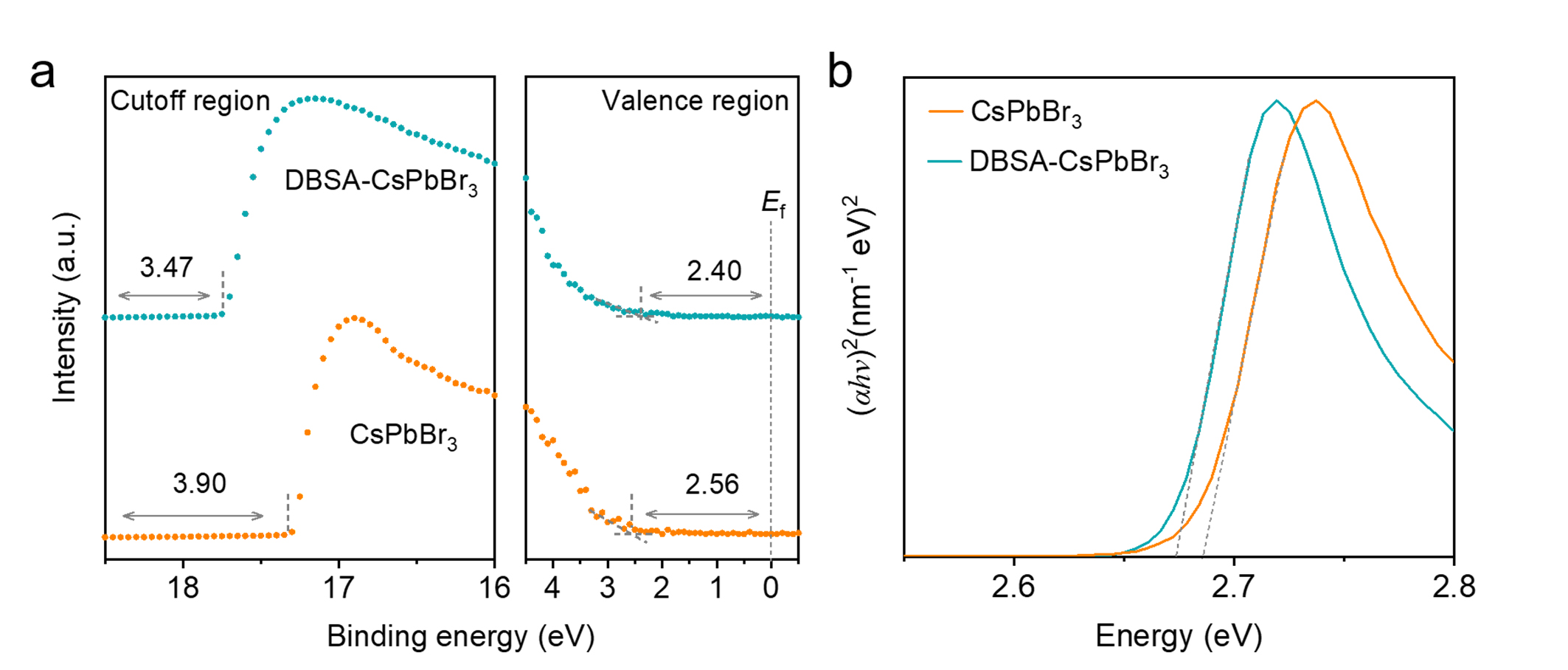


**Fig. S14:** (a) UPS spectra for the cutoff (left) and valence (right) regions, and (b) Tauc plots for CsPbBr_3_ and DBSA-CsPbBr_3_ NPLs. According to the relationship between the absorption coefficient (*α*) and optical bandgap (*E*_g_), namely, (*αhν*)^2^ = A (*hν* － *E*_g_), where *h* is the Planck constant and *ν* is the frequency, the Tauc plots were used to calculate the bandgaps of CsPbBr_3_ and DBSA-CsPbBr_3_ NPLs.


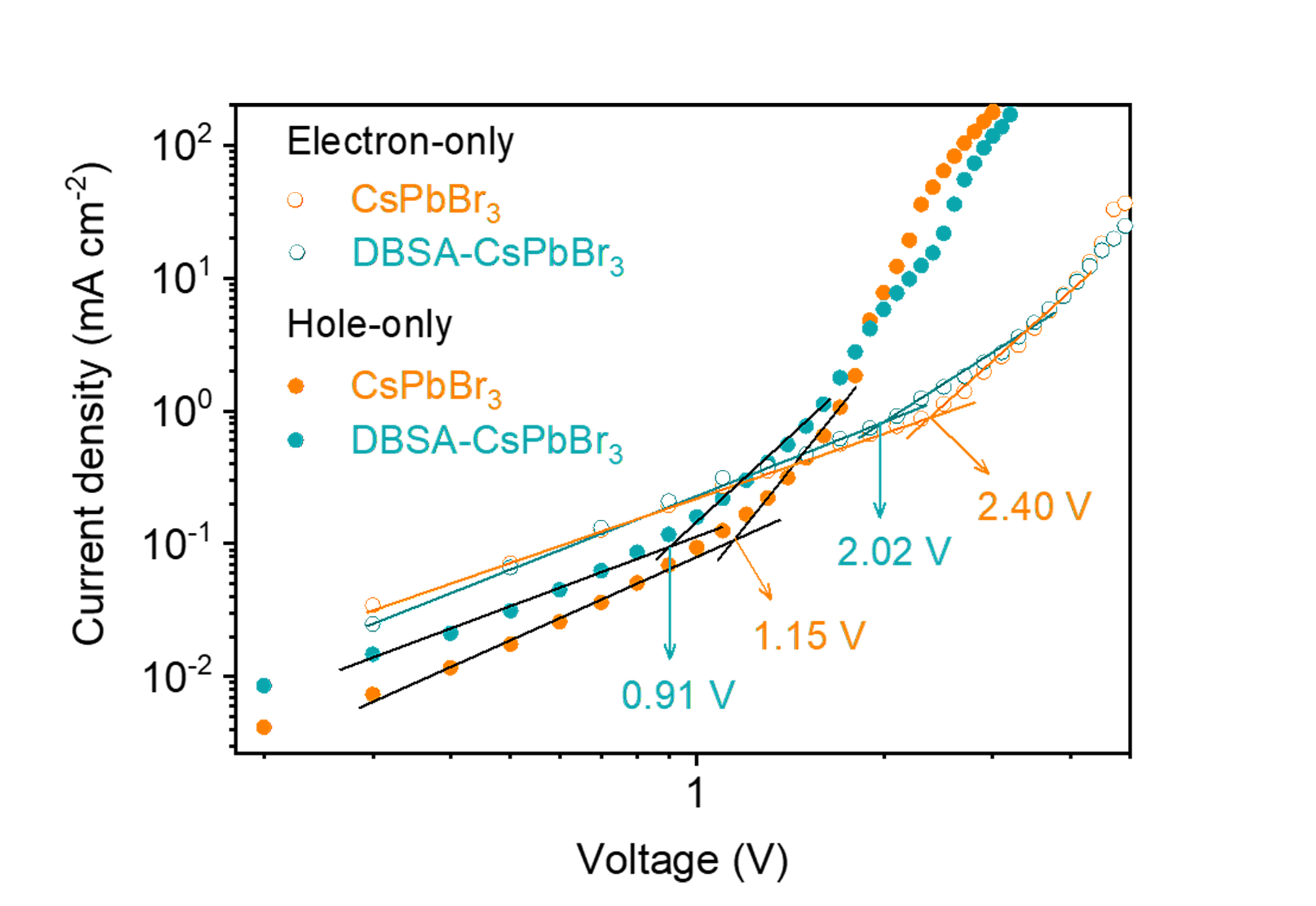


**Fig. S15:** *J−V* curves of the “electron-only” devices (ITO/NPLs/TPBi/POT2T/Al) and “hole-only” devices (ITO/PEDOT:PSS/Poly-TPD/PEI/NPLs/MoO_3_/Ag) based on CsPbBr_3_ and DBSA-CsPbBr_3_ NPLs.


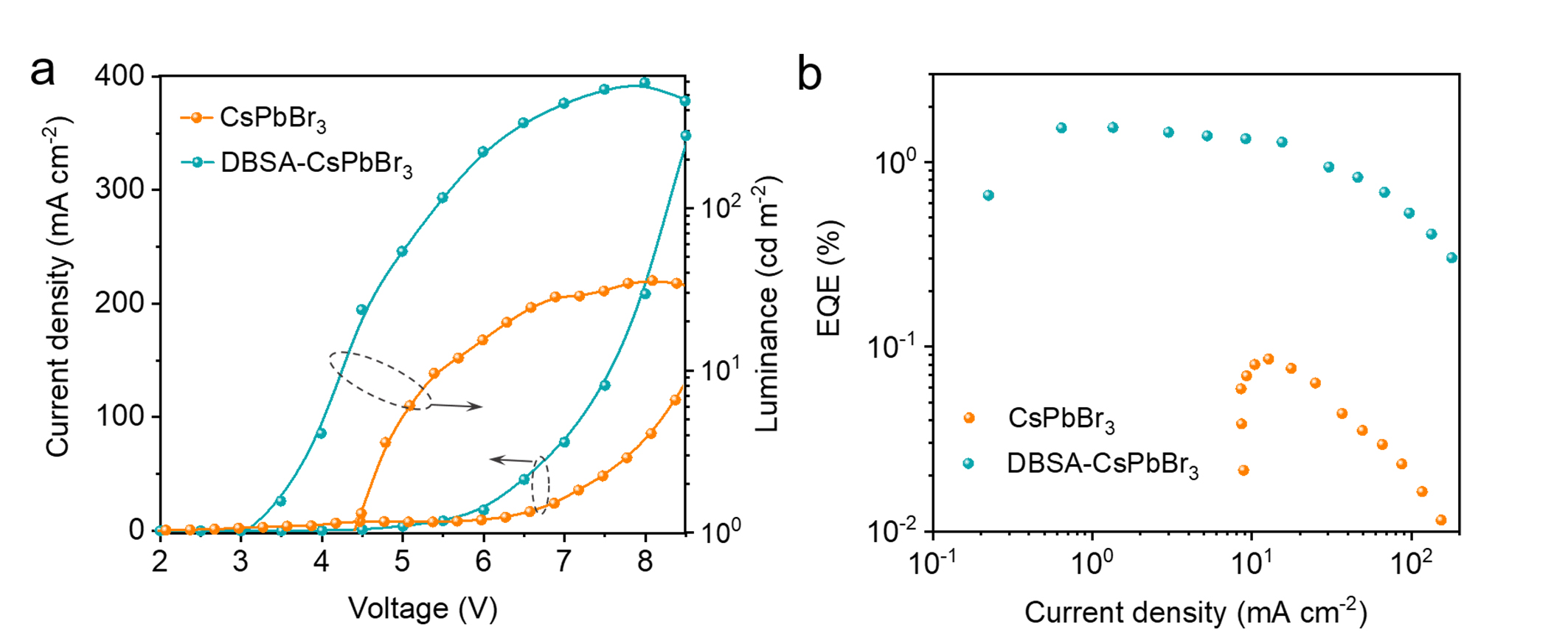


**Fig. S16:** (a) *J*–*V*–*L* curves and (b) EQE‒*J* plots of LEDs based on CsPbBr_3_ and DBSA-CsPbBr_3_ NPLs.


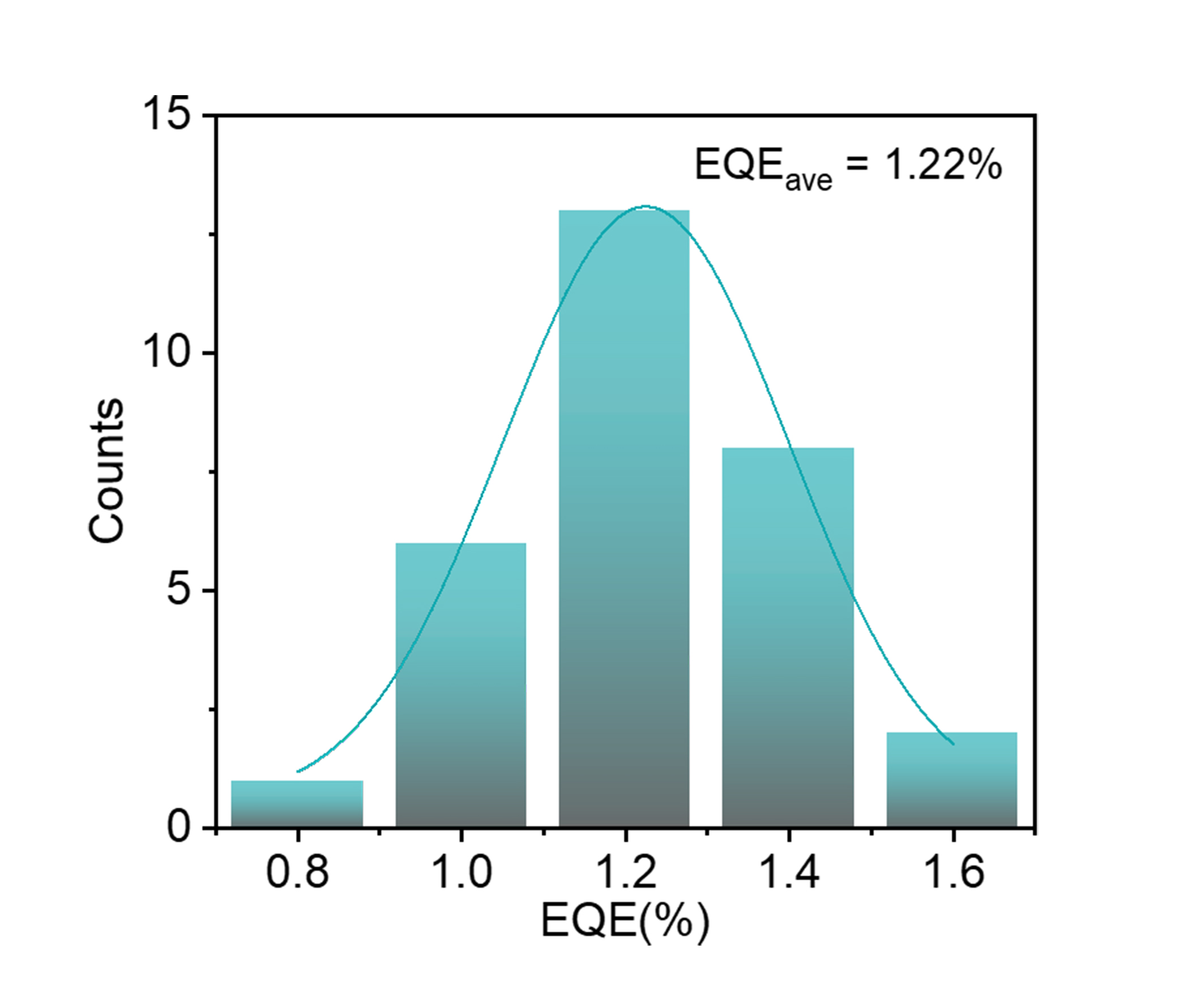


**Fig. S17:** EQE histogram for 30 DBSA-CsPbBr_3_ NPL-based LEDs.


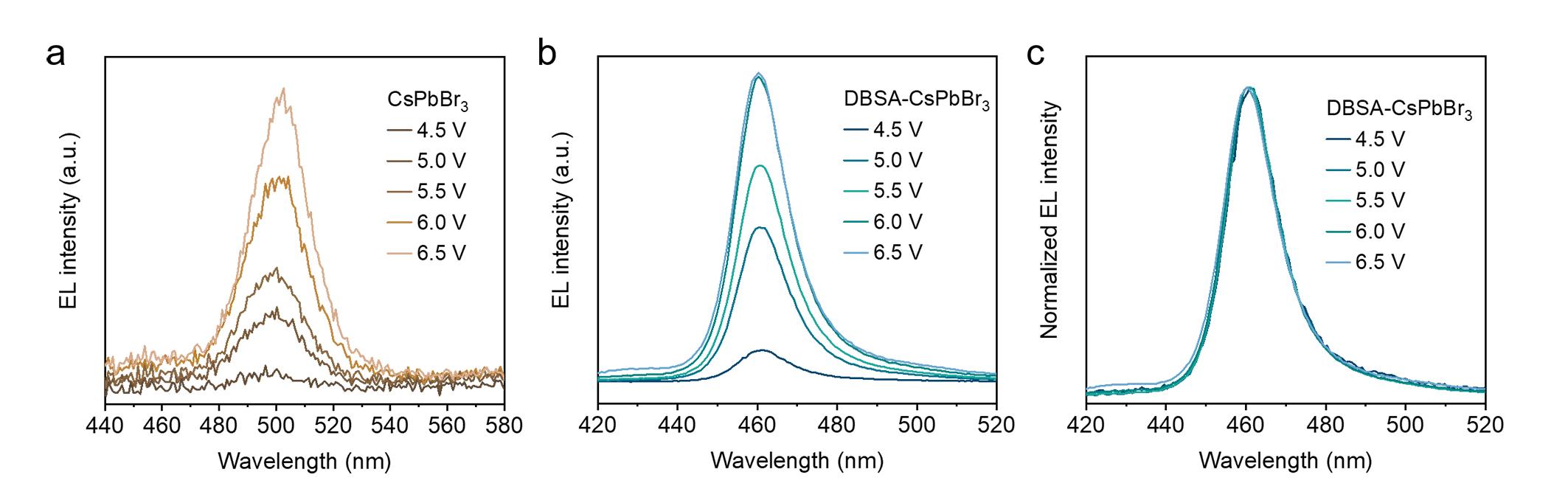


**Fig. S18:** EL spectra of LEDs based on (a) CsPbBr_3_ and (b) DBSA-CsPbBr_3_ NPLs measured at various biases. (c) The normalized EL spectra of DBSA-CsPbBr_3_ NPL-based LEDs at various biases.

**Supplementary Tables**

**Table S1:** Summary of the gain and loss of the electrons between Cs and O atoms obtained by Bader charge calculation.

| Atom | O1 | Cs1 | O2 | Cs2 | O3 | Cs3 |
| --- | --- | --- | --- | --- | --- | --- |
| Δ*е* | +0.94 | -0.88 | +0.94 | -0.88 | +0.93 | -0.87 |
| Bond length (Å) | 2.91 | | 3.25 | | 3.11 | |

**Table S2:** Fitting results of TRPL decay curves.

| Sample | *τ*_1_ (ns) | *f*_1_ (%) | *τ*_2_ (ns) | *f*_2_ (%) | *τ*_ave_ (ns) | R^2^ |
| --- | --- | --- | --- | --- | --- | --- |
| **CsPbBr_3_ NPL** | 0.9 | 52 | 2.7 | 48 | 1.8 | 0.99 |
| **DBSA-CsPbBr_3_ NPL** | 4.0 | 100 |  |  | 4.0 | 0.99 |

Note: The fitting equation is *I*_TRPL_(t) =Σ*A*_i_exp(−*τ*/*τ*_i_), where *A*_i_ represents the amplitude, and *τ*_i_ is the constant for PL decay components, respectively. A bi-exponential fit has been used for CsPbBr_3_ NPLs; the two components are assigned to radiative recombination (short lifetime *τ*_1_) and trap-mediated recombination (long lifetime *τ*_2_). A mono-exponential fit could be used for DBSA-CsPbBr_3_ NPLs, indicating trap-free radiative recombination.

**Table S3:** The calculating trap density (*n*_trap_) of CsPbBr_3_ and DBSA-CsPbBr_3_ NPLs.

| Sample | *n*_trap_ (electron-only) | *n*_trap_ (hole-only) |
| --- | --- | --- |
| **CsPbBr_3_ NPL** | 4.24×10^19^ cm^-3^ | 2.03×10^19^ cm^-3^ |
| **DBSA-CsPbBr_3_ NPL** | 3.56×10^19^ cm^-3^ | 1.61×10^19^ cm^-3^ |

Note: The trap density (*n*_trap_) can be calculated as *n*_trap_ = 2*εε_0_V*_TFL_/*eL*^2^, where *e* is the elementary charge (1.602×10^-19^ C), and *ε*, *ε_0_* and *L* are the relative dielectric constant (23), the vacuum permittivity (8.854×10^-12^ F m^-1^), and the thickness of the perovskite film (12 nm), respectively.
